# Advances in extracellular vesicle-based nanomedicine for regenerative orthopaedics

**DOI:** 10.1186/s12951-025-03906-w

**Published:** 2025-12-14

**Authors:** Sara Gil Izquierdo, Andrés Fernández Pilar, Jaqueline Lourdes Rios, Khoon S. Lim, Wei Seong Toh, Chaozong Liu, Mario Gimona, Debby Gawlitta, Kenny Man

**Affiliations:** 1 Department of Oral and Maxillofacial Surgery & Special Dental Care, University Medical Center Utrecht, Utrecht, The Netherlands; 2 Regenerative Medicine Center Utrecht, Utrecht, The Netherlands; 3 Department of Orthopaedics, University Medical Center Utrecht, Utrecht, The Netherlands; 4 School of Medical Sciences, University of Sydney, Sydney, NSW Australia; 5 Department of Orthopaedic Surgery, Yong Loo Lin School of Medicine, National University of Singapore, Singapore, Singapore; 6 Institute of Orthopaedic & Musculoskeletal, Division of Surgery & Interventional Science, University College London, Royal National Orthopaedic Hospital, Stanmore, London, UK; 7 Good Manufacturing Practice Laboratory, Paracelsus Medical University, Salzburg, Austria; 8 Research Program “Nanovesicular Therapies”, Paracelsus Medical University, Salzburg, Austria; 9 Ludwig Boltzmann Institute for Nanovesicular Precision Medicine, Salzburg, Austria

**Keywords:** Extracellular vesicles, Nanomedicine, Bone, Cartilage, Osteoarthritis, Regenerative medicine, Orthopaedics

## Abstract

The increasing prevalence of bone and cartilage injuries presents a persistent clinical challenge in orthopaedic medicine. Current standard-of-care approaches and regenerative strategies, including cell-based therapies and conventional biomaterials, remain suboptimal due to their limited capacity to precisely modulate the complex cellular and molecular mechanisms governing cartilage repair, bone remodelling, and osseointegration. Extracellular vesicles (EVs), nature’s nanoscale mediators of intercellular communication, have recently emerged as powerful bioactive entities with immense potential to orchestrate tissue regeneration. This review critically examines the diverse and dynamic roles of EVs in orthopaedic repair, emphasizing their mechanistic contributions to osteogenesis, chondrogenesis, and osteochondral interface regeneration. We further highlight recent advances in bioengineering approaches designed to enhance EV therapeutic efficacy, including surface functionalization, cargo engineering, and biomaterial-based delivery systems. Finally, we discuss the advent of EV-mimetic nanoplatforms as next-generation therapeutics, underscoring their translational potential to overcome current clinical limitations. Collectively, this review highlights the transformative promise of EV-based and EV-inspired nanotechnologies in advancing the frontier of bone and cartilage regenerative medicine.

**Graphical abstract:**

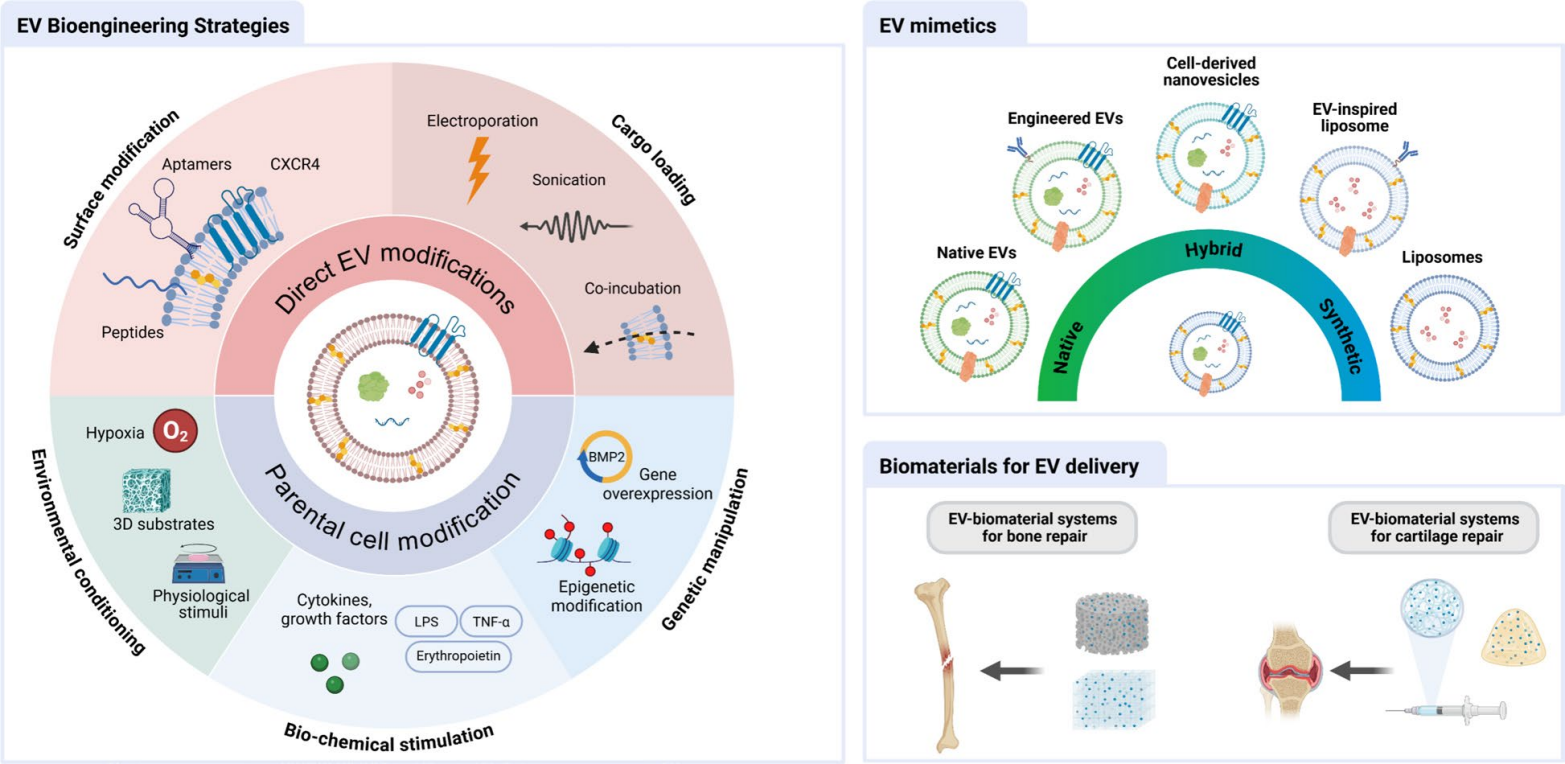

## Introduction

With the globally growing aging population, the incidence of bone and cartilage injuries and associated disorders has increased substantially, affecting an estimated of 1.71 billion people worldwide, including those with fractures, osteoarthritis (OA) and lower back pain [1]. Degenerative conditions such as OA cause pain, functional limitations, and joint deformities, imposing substantial burdens on healthcare systems and significantly diminishing patients’ quality of life [2]. Current management relies on conservative measures, including corticosteroid injections, non-steroidal anti-inflammatory drugs, physical therapy, or lifestyle changes [3]. Surgical procedures including mosaicplasty and microfracture, provide palliative benefits effects but often yield suboptimal long-term outcomes [2, 4, 5]. Total joint replacement remains the terminal option, effectively relieving pain but carrying risks of complications, implant failure, and revision surgery, particularly in younger patients [6, 7]. Similarly, bone disorders like osteoporosis are a major health concern, with an osteoporotic fracture occurring globally every three seconds [8]. Conventional treatments for bone repair, such as autologous and allogeneic bone grafting, are often associated with donor site morbidity, risks of infection, and limited availability [9, 10]. these challenges underscore the persistent clinical need for innovative strategies to repair and regenerate damaged bone and cartilage.

Given the limitations of current treatments, there has been extensive research focused on biomaterial- and cell-based tissue engineering approaches to repair damaged bone and cartilage [11, 12]. Despite their promise, biomaterial systems for bone and cartilage repair face significant hurdles. For bone regeneration, key limitations include achieving proper mechanical matching to prevent stress shielding, promoting true osseointegration instead of fibrous encapsulation, precisely controlling degradation rates, and mitigating infection risks [13, 14]. In cartilage repair, biomaterials struggle to replicate the complex mechanical properties and zonal architecture of native hyaline cartilage and achieve stable integration with host tissue, while also maintaining chondrocyte viability in an avascular environment [15, 16]. Regenerative approaches harnessing mesenchymal stem/stromal cells (MSCs) have garnered tremendous interest for musculoskeletal regeneration due to their multilineage potential and wide availability [17, 18]. Although MSC-based therapies have shown promise in regenerative orthopaedics, the translation of cell-based therapies is hindered by issues associated with low cell survival rates, regulatory hurdles, high manufacturing costs, ethical concerns, and risks such as tumour formation [19, 20]. Interestingly, growing evidence suggests that the bioactive factors secreted by MSCs play a key role in their therapeutic effects, prompting researchers to explore the use of these cell-derived trophic factors for regenerative applications [21, 22].

In recent years, there has been a growing number of studies demonstrating the influence of the cell’s secretome in mediating key cellular functions [23, 24]. These studies have highlighted the important role of extracellular vesicles (EVs), cell-secreted lipid nanoparticles, on intercellular communication and tissue regeneration. By harnessing the regenerative potential of EVs, researchers aim to overcome many limitations of conventional cell- and biomaterial-based therapies, positioning EVs as a central focus of next-generation musculoskeletal regenerative strategies.

## Extracellular vesicles: nature’s nanosized messengers

**Fig. 1:**
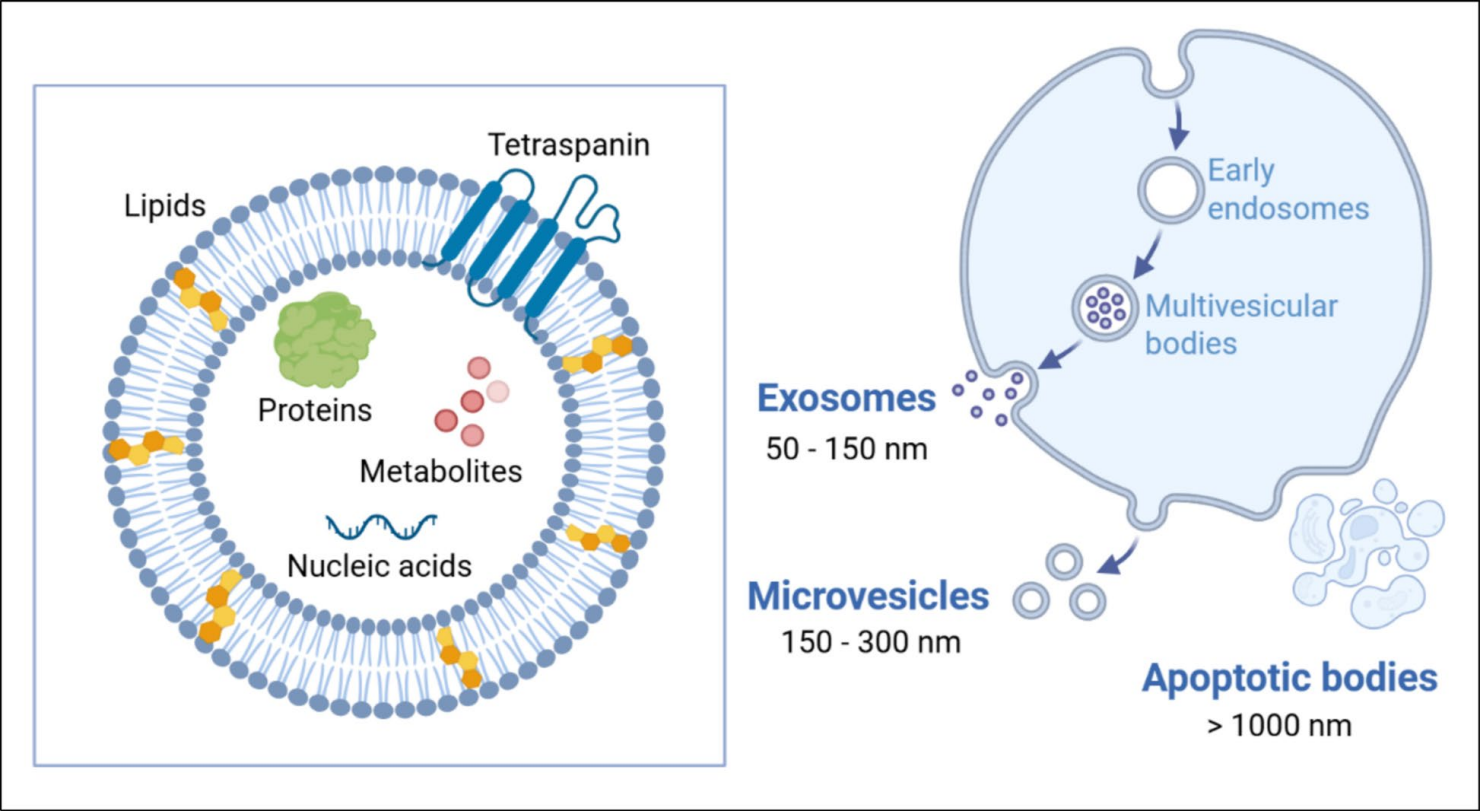
EV classification, composition, and biogenesis. overview of the major EV subtypes, differentiated by their biogenesis pathways and characteristic size ranges. exosomes (50–150 nm) originate from the endosomal pathway, forming within multivesicular bodies that subsequently fuse with the plasma membrane. microvesicles (150–300 nm) bud directly from the plasma membrane. apoptotic bodies (>1000 nm) are larger vesicles released during programmed cell death. These distinct modes of formation contribute to their varied compositions and biological roles in intercellular communication. insert shows a schematic illustration of a typical EV, highlighting its fundamental components. EVs are enveloped by a lipid bilayer membrane, often adorned with transmembrane proteins such as tetraspanins (e.g., CD9, CD63, CD81). their diverse internal cargo includes various proteins, lipids, metabolites, and nucleic acids (mRNA, miRNA, DNA), reflecting the cellular state of their origin

EVs are nanosized lipid-based particles that carry a diverse bioactive cargo of proteins, metabolites, and nucleic acids [25] (Fig 1). These vesicles are typically classified into exosomes (50–150 nm), microvesicles (150–300 nm), and apoptotic bodies (>1000 nm), each with distinct biogenesis, cargo, and size characteristics [26]. Exosomes are formed via the endosomal pathway and are secreted from the plasma membrane upon their fusion with multivesicular bodies [27]. Microvesicles are formed through outward blebbing of the plasma membrane [28], and apoptotic bodies are produced from the plasma membrane when cells undergo programmed cell death [29]. Once in the extracellular space, EVs can interact with target cells through multiple mechanisms that enable them to deliver their bioactive cargo and modulate cellular functions. They can bind to cell surface receptors via specific ligand-receptor interactions, triggering signalling pathways without being internalized. Moreover, a particular subset of EVs known as matrix vesicles, can bind the extracellular matrix (ECM), providing a reservoir of extracellular growth factors [30]. Alternatively, EVs can be taken up by target cells through various endocytic processes, or directly fuse with the plasma membrane, releasing their contents into the cytoplasm. These interactions allow EVs to influence gene expression and modulate cell response, making them promising tools for therapeutic applications [31].

Various isolation methods, ranging from ultracentrifugation, filtration and size-exclusion chromatography to polyethylene glycol/polymer-based enrichment and antibody-based methods, have been used for EV isolation. However, isolation of specific EV subtypes is presently challenging due to their overlapping biochemical and biophysical properties, and the lack of definitive markers to unambiguously identify an EV subtype. In view of these challenges, the collective term "EVs" is used throughout this review in accordance with the Minimal Information for Studies of Extracellular Vesicles (MISEV) 2023 guidelines [32] to refer to the heterogeneous population of vesicles released by cells.

EVs are involved in key physiological processes such as the maintenance of homeostasis and the regulation of cellular functions [33]. Several studies have reported the importance of these EV-associated bioactive factors in intercellular communication to regulate biological behaviour [34]. The advances in the EV field, emphasise their potential influence on future healthcare applications [31]. Thus, the use of these cell-derived nanoparticles has attracted interest as potential acellular therapy for regenerative orthopaedics [35–37]. Employing these naturally derived nanoparticles presents numerous advantages such as a lower immunogenicity, high physiochemical stability, ease of storage and off-the-shelf availability compared to cell-based therapies [31, 38]. Moreover, there is increasing evidence reporting the comparable or even superior regenerative capacity of these EVs compared to their parent cells. For example, in a rat tibial distraction osteogenesis model, Jia et al. reported that EVs from endothelial progenitor cells (EPCs) induced a similar degree of bone regeneration compared to the EPC-treated group [39]. Furthermore, Zavatti et al. demonstrated that EVs derived from human amniotic fluid stem cells showed greater therapeutic potential in a rat model of OA than the stem cells themselves, as indicated by improved pain tolerance and more effective cartilage regeneration [40].

In addition to their diverse cargo that produces a broad therapeutic effect, EVs have a complex surface molecular composition that enhances their ability to target tissues, primarily through membrane-level interactions (Fig 1) [31]. Moreover, this biological diversity gives EVs therapeutic advantages over synthetic nanoparticles, as they naturally exhibit biocompatibility, low immunogenicity, and high physicochemical stability [41, 42]. These characteristics not only improve their regenerative potential but also make them suitable candidates for drug carriers [43, 44], leading to a growing interest in these naturally derived nanoparticles as promising nanoscale therapeutics for regenerative orthopaedics.

## The role of EVs in bone and cartilage regeneration

Accumulating evidence underscores the intrinsic involvement of EVs in intercellular communication, critically regulating bone [35, 45, 46] and cartilage [47–49] development and homeostasis. A comprehensive understanding of the fundamental mechanisms, by which native EVs mediate tissue development and regeneration, holds significant promise for leveraging these vesicles in regenerative medicine strategies. While native EVs may influence a variety of bone- and cartilage-related pathologies, the focus of this review is on their role in bone and cartilage repair and regeneration. Investigation of their broader therapeutic potential in other conditions, such as inflammatory or metabolic skeletal disorders, falls outside the scope of the present article.

### Native EVs in bone regeneration

EVs have emerged as critical mediators of intercellular communication in bone regeneration. Their ability to shuttle a diverse cargo of proteins, lipids, and nucleic acids, including epigenetic regulators, allows them to orchestrate the complex and temporally coordinated processes necessary for successful bone repair [50]. Within this intricate landscape, EVs play distinct and interconnected roles in promoting osteogenesis, vascularization, immunomodulation, and osteoclastogenesis, orchestrated by the multiple cell types within the bone fracture niche [51].

#### MSC-derived EVs

MSCs are central orchestrators of fracture repair through a multifaceted interplay of immunomodulation, trophic factor secretion, and EV-mediated signalling [52, 53]. During the initial inflammatory phase, MSCs help control excessive inflammation by releasing anti-inflammatory cytokines and chemokines. This activity shapes the immune environment at the fracture site and promotes the critical transition of macrophages from the pro-inflammatory M1 state to the anti-inflammatory M2 state, thereby resolving inflammation and preparing the tissue for repair [54]. Subsequently, MSCs secrete a repertoire of trophic factors that stimulate angiogenesis and recruit osteoprogenitor cells, promoting bone formation and matrix remodelling. In the later stages, MSCs contribute to skeletal homeostasis by modulating the balance between osteoblast and osteoclast activity [55]. Studies have reported that MSC EVs are critical regulators of ossification and bone homeostasis [45]. Specific microRNAs (miRs) encapsulated within these vesicles have been shown to promote osteogenic differentiation and bone regeneration through diverse mechanisms, including the stabilization of key osteogenic transcription factors (*e.g.*, Runx2) and the activation of signalling pathways (*e.g.*, Wnt/β-catenin) [56, 57]. Yang et al. demonstrated that EVs derived from bone marrow MSCs (BMSCs), particularly those containing miR-29b-3p, significantly enhance regeneration in a mouse femoral fracture model [58]. Mechanistically, these miR-29b-3p-enriched EVs promote bone repair by modulating the PTEN/PI3K/AKT signalling pathway, thereby facilitating cell proliferation, survival, and osteogenic differentiation that are essential for bone regeneration. Similarly, Jiang et al. revealed that BMSC-derived EVs promoted fracture healing by delivering miR-25 [59]. EV-associated miR-25 directly targets and suppresses SMURF1, an E3 ubiquitin ligase, thereby preventing the ubiquitination and degradation of Runx2. By stabilizing Runx2, miR-25 promotes osteogenic differentiation and accelerates fracture healing in mice, highlighting its role as a key EV cargo in bone regeneration. Beyond osteogenesis, MSC EVs also orchestrate angiogenesis, a crucial process for fracture healing. For example, BMSC-EVs containing miR-29a promote endothelial cell migration and proliferation, while the EV-mediated transfer of proteins like Nidogen1 can also enhance angiogenesis and improve bone defect repair when delivered locally from a biomaterial scaffold [60]. Similarly, Zhang et al. showed that compared to the non-treated control, BMSC EV treatment enhanced fracture healing in a rat non-union model [61]. These EVs led to improved bone volume, fracture-end connectivity, and mineral content, by stimulating both osteogenesis and angiogenesis, likely via BMP-2/Smad1/Runx2 and HIF-1α/VEGF pathways. As earlier mentioned, the transition from M1 to M2 macrophages is essential for resolving inflammation and promoting tissue repair. On this note, Chuah et al. demonstrated in a rat calvarial defect model that MSC-EVs enhance bone healing by modulating macrophage polarisation toward [62] a pro-regenerative M2 phenotype. This immunomodulatory effect, together with the stimulation of angiogenesis and osteogenesis, reduced inflammation and significantly enhanced cellular infiltration, vascularization, and mineralization, thereby promoting overall bone regeneration [62].

#### Osteoblast-derived EVs

Osteoblasts are the primary cells responsible for bone formation, playing a crucial role in developing skeletons, continuous bone remodelling throughout life, and the repair of damaged bone [63]. Research has begun to elucidate the role of osteoblast-derived EVs in driving the mineralisation of the ECM. Studies have reported that pre-osteoblast EVs were able to stimulate the osteogenic differentiation of hBMSCs *in vitro* [35, 64]. Osteoblasts are also known to secrete matrix-bound vesicles, which play an important role in stimulating mineralization. Matrix vesicles were found to be enriched in tissue non-specific alkaline phosphatase and annexin proteins, which have been linked to ECM mineralization [65, 66]. Su et al. demonstrated that bone tissues obtained from osteoporotic mice exhibited a lower quantity of matrix vesicles, associated with reduced bone mineral density [67]. Mizukami et al. isolated matrix vesicles from murine osteoblasts and delivered them in a gelatin hydrogel to a mouse femoral bone defect model, resulting in enhanced bone repair [68]. Interestingly, recent evidence has highlighted the role of osteoblast EVs in inhibiting bone formation [69]. For instance, Uenaka et al. found that EVs from mature osteoblasts inhibited bone formation and promoted osteoclastogenesis, suggesting their role in shifting bone remodelling from formation to bone resorption [46]. Similarly, Deng et al. showed that osteoblast EVs contain and transfer receptor activator of nuclear factor kappa-B ligand (RANKL) protein to osteoclast precursors [70]. This direct transfer of RANKL then stimulates the RANKL-RANK signalling pathway, thereby facilitating osteoclast formation. Similarly, Uenaka et al. showed that mature osteoblast-derived EVs inhibited bone formation and enhanced osteoclastogenesis, indicating the capacity of these vesicles to trigger the transition from bone formation to its resorption [46]. Collectively, these studies highlight the diverse roles of osteoblast EVs in the bone microenvironment.

#### Osteoclast-derived EVs

Osteoclasts, specialized multinucleated cells derived from the hematopoietic cell lineage, are the primary mediators of bone resorption. These potent bone-degrading cells secrete acid to dissolve the mineralized ECM, and highly active proteolytic enzymes, to degrade collagen and other organic matrix proteins during bone remodelling [71, 72]. EVs released from these cells have been reported to serve as a critical direct negative regulator of bone formation. For example, Li et al. demonstrated that osteoclast EVs inhibit osteoblastic bone formation via the transfer of miR-214-3p [73]. This EV miRNA directly targets and suppresses the expression of osteogenic genes in osteoblasts, thereby reducing bone formation. Similarly, Yang et al. reported that osteoclast EVs enriched with miR-23a-5p actively inhibit osteogenesis [74]. This was achieved by downregulating Runx2 and altering YAP1 signalling, which together alleviate the suppression of metallothionein 1D pseudogene (MT1DP). This intricate cascade ultimately disrupts the gene expression essential for proper osteoblast maturation and bone formation. Conversely, Liang et al. described that osteoclast EVs containing miR-324 directly induce MSCs’ osteogenic differentiation by inhibiting ARHGAP1, a key negative regulator of osteogenesis [75]. These studies reveal the intricate roles of EVs in mediating communication between osteoclasts and osteoblasts, highlighting a crucial regulatory pathway in bone remodelling.

#### Osteocyte-derived EVs

Osteocytes, which are terminally differentiated osteoblasts reside embedded within the mineralized bone matrix. They play a crucial role in regulating bone homeostasis [76]. They modulate osteoblast and osteoclast function through paracrine signalling, notably by secreting RANKL, which promotes osteoclastogenesis, and osteoprotegerin (OPG), a RANKL decoy receptor that inhibits osteoclast differentiation [77]. Osteocytes also play an important role in bone mechanotransduction by altering their signalling profiles in response to mechanical loading. Recent studies have highlighted the role of osteocyte-derived EVs in modulating osteogenesis. For example, Morrell et al. demonstrated that EVs from mechanically strained osteocytes, through shear stress, stimulated osteogenic differentiation of human periodontal ligament stem cells via the miR-181b-5p/PTEN/AKT signalling pathway [78]. Wang et al. investigated the influence of age on biological function of EVs secreted from osteocytes [79]. In this study, EVs were isolated from primary osteocytes, obtained from either young (2-month-old) or old mice (16-month-old). The authors showed that young osteocytes promote bone formation by releasing EVs containing tropomyosin-1 (TPM1). These young osteocyte EVs significantly enhance osteogenesis *in vitro* and increase bone mass and strength *in vivo* when compared to old osteocyte EVs. Mechanistically, TPM1 transferred via EVs increases BMSC intracellular F-actin polymerization, thereby driving osteogenesis. This finding highlights the crucial role of osteocyte-derived EVs and TPM1 in bone homeostasis and suggests their potential as therapeutic targets for age-related bone loss.

#### Immune cell-derived EVs

Immunomodulation is a crucial process in regulating bone fracture healing [51, 80], with EVs shown to be integral mediators of immune regulation. Macrophages, which are key players of the immune system, are central to the host defence, tissue homeostasis, and regeneration [81]. Their remarkable plasticity allows them to dynamically respond to microenvironmental cues, modulating biological processes through the secretion of trophic factors including EVs. Kang et al. studied how macrophage polarisation affects the therapeutic potential of macrophage-derived EVs. In a rat calvarial defect model, they found that EVs from M1 macrophages inhibited bone repair, while EVs from M0 and M2 macrophages promoted bone regeneration [82]. Similarly, Wang et al. demonstrated that M2-EVs modulate the osteoimmune microenvironment in diabetic fractures, accelerating healing by decreasing pro-inflammatory cells and inducing M1 to M2 macrophage conversion via the PI3K/AKT signalling pathway [83]. Dendritic cells are key antigen-presenting cells that bridge the innate and adaptive immune systems by capturing, processing, and presenting antigens to T cells, thereby initiating immune responses. Elashiry et al. demonstrated that dendritic cell-derived EVs engineered to carry TGF-β1 and IL-10, mitigated inflammatory alveolar bone loss in a murine periodontitis model [84]. This therapeutic effect was achieved through immunomodulation, characterized by enhanced regulatory T cell recruitment and suppressed Th17 differentiation, ultimately leading to a reduction in osteoclast-mediated bone resorption.

#### Endothelial cell-derived EVs

Vascularization of bone tissue is a crucial process for effective healing, as an adequate nutrient supply is essential for the repair of critical-sized large bone defects [85, 86]. It has been reported that EVs not only directly stimulate bone formation but also enhance vascularization of the newly formed tissue [51]. Recent studies have highlighted the diverse functions of endothelial cell-derived EVs in bone regeneration. For instance, Cui et al. showed that EPC-derived EVs, naturally enriched with LncRNA-MALAT1, played a dual role: they enhanced macrophage migration and promoted osteoclastic differentiation by sequestering miR-124, a known negative regulator of osteoclastogenesis [87]. These EVs ultimately facilitated bone repair in a mouse femur fracture model by boosting the recruitment and differentiation of osteoclast precursors. Similarly, Jia et al. showed that EPC-derived EVs accelerate bone regeneration during distraction osteogenesis in rats by primarily stimulating angiogenesis, leading to increased vessel density and enhanced overall bone formation and consolidation [39]. *In vitro*, these EVs promoted endothelial cell proliferation, migration, and angiogenic capacity, confirming their pro-angiogenic role. Collectively, this growing body of evidence highlights the diverse roles that native EVs from various cells within the bone microenvironment play in regulating bone repair (Table 1).

**Table 1:** Overview of studies demonstrating the role of native EVs in bone repair

EV source	Cargo	Study findings	Ref
MSCs	miR-29b-3p	MSC EVs enhance bone repair by modulating the PTEN/PI3K/AKT signalling axis	[58]
	Nidogen1	MSC EVs promote angiogenesis and improve bone defect repair	[60]
	N/A	Activate osteogenic differentiation and angiogenesis, via pathways such as BMP-2/Smad1/RUNX2 and HIF-1α/VEGF	[61]
Osteoblasts	miR-143-3p	Mature osteoblast-derived EVs inhibited bone formation and enhanced osteoclastogenesis	[46]
	N/A	Osteoblast-derived matrix vesicles promote femoral defect repair	[68]
Osteoclasts	miR-214-3p	Osteoclast differentiation can inhibit osteoblastic bone formation via the PTEN/PI3K/AKT pathway	[73]
	miR-324	osteoclast EVs stimulate MSCs osteogenic differentiation by inhibiting ARHGAP1	[75]
Osteocytes	N/A	Mechanically strained osteocytes EVs stimulated osteogenic differentiation via the miR-181b-5p/PTEN/AKT signalling pathway	[78]
	TPM1	Young osteocytes EVs enhance osteogenesis *in vitro* and increase bone mass and strength *in vivo*	[79]
Macrophages	N/A	M2-EVs modulate osteoimmune microenvironment in diabetic fractures and accelerate fracture healing	[83]
	M1 EVs (miR-155), M2 EVs (miR-378a)	EVs from M1 macrophages inhibited bone repair whereas both M0- and M2-derived EVs promoted bone regeneration	[82]
Endothelial cells	miR-126	EPC EVs enhanced bone formation and consolidation, accompanied by increased vessel density	[39]
	LncRNA-MALAT1	Facilitated repair in a mouse femur fracture model by boosting the recruitment and differentiation of osteoclast precursors	[87]

### Native EVs in cartilage regeneration

Articular cartilage damage often involves chondrocyte stress, ultimately leading to inflammation, ECM degradation, and chondrocyte apoptosis [88–90]. A deeper understanding of the pathogenetic mechanisms underlying cartilage injury is crucial for the development of novel therapeutic strategies, aimed at restoring tissue homeostasis. Although EVs are known to be present in both articular cartilage and the growth plate, their exact physiological functions have long remained unclear, a challenge common to much of EV biology [47]. Chondrocyte-derived EVs, initially identified as ‘calcifying globules’ in 1970 [91], are implicated in both cell-to-cell signalling and the mineralization of cartilage tissue through the promotion of hydroxyapatite crystal deposition. This role is further supported by studies in a rat model of temporomandibular joint OA, where autophagosome-derived EVs were shown to trigger cartilage calcification under pathological conditions [92].

#### MSC-derived EVs

Recent research has begun to elucidate the potential of native EVs to promote cartilage regeneration. Casanova et al. demonstrated that human articular chondrocyte- and BMSC-derived EVs, when immobilized on a nanofibrous substrate, induced a chondrogenic phenotype in BMSCs more effectively than standard chondrogenic differentiation medium, as evidenced by the enhanced expression of cartilage-related genes [93]. Consistent with these findings, Hosseinzadeh et al. reported that both MSC- and chondrocyte-derived EVs stimulated chondrogenesis, with MSC-derived EVs exhibiting greater potential at a concentration of 100 μg/ml [94]. In addition to promoting chondrogenesis, MSC EVs have been shown to exert protective effects on existing chondrocytes. Liu et al. found that MSC EVs increased GIT1 expression, leading to enhanced chondrocyte proliferation and reduced apoptosis, via the lncRNA-KLF3-AS1/miR-206/GIT1 axis [95]. Similarly, Zhang et al. demonstrated that MSC EVs were efficacious in promoting osteochondral regeneration in rats, and this was mediated through a well-orchestrated mechanism of augmenting cellular proliferation, attenuating apoptosis, increasing matrix synthesis, and enhancing preferential M2 over M1 macrophage infiltration with concomitant suppression of synovial inflammation [96]. Of these EV-mediated activities, MSC EVs enhanced chondrocyte proliferation and migration partly through CD73-mediated adenosine activation of AKT and ERK signalling pathways [96]. The ability of MSC EVs to promote cell proliferation and migration has been corroborated by other studies as well [95, 97]. Given the critical role of ECM degradation in OA pathogenesis [98], the influence of EVs on ECM homeostasis is of particular interest. Tofino-Vian et al. investigated the effects of adipose-derived MSC (ADSC) EVs on OA chondrocytes, observing a downregulation of inflammatory (TNF-α, IL-6, PGE2, and NO) and catabolic (MMP-13) mediators, coupled with an upregulation of collagen II production [99]. Zhao et al. identified a novel mechanism, by which human umbilical cord MSC EVs (hUC-MSC-EVs) alleviate knee OA [100]. The authors showed that hUC-MSC-EVs interact with METTL3, a methyltransferase, to reduce the N6-methyladenosine (m6A) modification of NLRP3 mRNA in macrophages. This subsequently suppresses NLRP3 inflammasome activation, leading to decreased secretion of pro-inflammatory factors and attenuated degradation of cartilage ECM. In a mouse model of OA, hUC-MSC-EVs effectively mitigated disease progression, highlighting a new therapeutic avenue for OA by modulating macrophage-mediated inflammation through an epigenetic mechanism involving the EV-METTL3-NLRP3-m6A axis. Zhao et al. reported that ADSC EVs upregulated miR-145 and miR-221 expression in periosteal cells, inhibiting H_2_O_2_-induced cell death and consequently enhancing proliferation and chondrogenic differentiation [101]. Furthermore, Chen et al. found that BMSC-derived EVs enriched with miR-136-5p enhanced *in vitro* chondrocyte migration and suppressed *in vivo* cartilage degeneration [102].

#### Synovial membrane MSCs

Zhu et al. reported that EVs derived from human synovial membrane MSCs (SMMSCs) enhanced chondrocyte migration and proliferation *in vitro* [103]. In a collagenase-induced OA mouse model, treatment with SMMSC EVs significantly improved cartilage repair, as evidenced by increased ICRS scores, reduced OARSI scores, and preservation of collagen type II content. Similarly, Qiu et al. showed that SMMSC EVs inhibited apoptosis and inflammatory responses by delivering miR-129-5p, which targets the 3’-UTR of high mobility group protein-1 to regulate IL-1β, thereby slowing OA progression [104].

Although MSC-derived EVs originate from the same cellular source, their regenerative effects on bone and cartilage may differ due to variations in their molecular cargo. Factors such as the physiological state of the parent MSCs, local environmental cues, and specific signaling pathways activated during EV biogenesis can influence the composition of EVs, thereby determining their tissue-specific regenerative outcomes [105, 106].

#### Cartilage cells-derived EVs

Beyond MSCs, EVs from cells of the chondrogenic lineage have also demonstrated their potential for OA treatment. For example, cartilage progenitor cells (CPCs) within the superficial zone of articular cartilage have been identified to exhibit strong recruitment and chondrogenic potential [107]. Moreover, compared to MSCs, CPCs have been reported to express lower levels of hypertrophic markers, such as collagen type X [108]. Feng et al. showed that CPC-derived EVs protect IL-1β-stimulated chondrocytes *in vitro* and, in a mouse model of posttraumatic OA, reduce ECM catabolism, inflammation, and cartilage degradation [109]. Likewise, Wang et al. reported that CPC EVs stimulated chondrocyte migration and proliferation *in vitro* and intra-articular injection ameliorated OA severity *in vivo*, likely due to the enrichment of miR-221-3p [110]. Li et al. showed that chondrocyte EVs delivered miR-8485 to BMSCs, which then directly inhibited the Wnt/β-catenin signalling pathway, a crucial step for initiating and progressing chondrogenesis [49]. Sang et al. demonstrated that intra-articular injection of chondrocyte-derived EVs, using a thermosensitive pluronic F-127-hyaluronic acid hydrogel, modulated M1 to M2 macrophage polarisation, thereby reducing inflammation and promoting ECM formation [111]. This shift in macrophage polarisation towards the M2 phenotype following intra-articular administration of primary chondrocyte-derived EVs in mice was also reported by Zheng et al. [112].

Large animal models are essential for evaluating cartilage repair as they closely mimic human joint size, biomechanics, and cartilage thickness, providing a more clinically relevant assessment of repair strategies than small animal models. Zhang et al. demonstrated the efficacy of MSC EVs for functional osteochondral repair in a clinically relevant porcine model [113]. Three weekly intra-articular injections of MSC EVs combined with hyaluronic acid significantly improved magnetic resonance imaging, macroscopic, and histological outcomes, enhanced biomechanical properties and increased subchondral bone volume, without adverse effects after 4 months.

In summary, native EVs derived from diverse cell types relevant to the joint microenvironment exhibit multifaceted roles in cartilage regeneration. They can promote chondrogenesis, protect chondrocytes from apoptosis, modulate inflammatory responses, influence ECM metabolism, and mediate these effects, at least in part, through EV-Cell interaction (Table 2). These findings underscore the therapeutic potential of harnessing the inherent signalling capabilities of native EVs to treat cartilage injuries and OA.

**Table 2:** Overview of studies demonstrating the role of EVs on cartilage repair

EV source	Cargo	Study findings	Ref
MSCs	KLF3-AS1	MSC EVs enhanced chondrocyte proliferation and reduced apoptosis, via the lncRNA-KLF3-AS1/miR-206/GIT1 axis	[95]
	Annexin A1	ADSC EVs reduced inflammatory and catabolic mediators, coupled with an upregulation of collagen II production	[99]
	miR-129-5p	SMMSC EVs inhibited apoptosis and inflammation by targeting the 3’-UTR of high mobility group protein-1	[104]
	N/A	SMMSC EVs improved cartilage repair in a collagenase-induced OA mouse model	[103]
	CD73	MSC EV mediated cartilage repair in osteochondral defects *in vivo* attributed to exosomal CD73-mediated adenosine activation of AKT and ERK signalling	[96]
CPCs	STAT3 regulatory proteins	CPC EVs exhibited protective effects on IL-1β-induced chondrocytes *in vitro* and inhibited ECM catabolism, and cartilage degradation *in vivo*	[109]
	miR-221-3p	Enhanced chondrocyte migration and proliferation *in vitro* and ameliorated OA severity *in vivo*	[110]
Chondrocytes	N/A	Regulation of macrophage polarization, thereby reducing inflammation and promoting ECM formation	[111]
	Mitochondrial proteins and proteins involved in immune processes	EVs prevented the development of OA by restoring mitochondrial dysfunction and stimulating M2 macrophage polarisation	[112]

## EV bioengineering strategies for regenerative orthopaedics

Despite the therapeutic promise of exploiting native EVs, limitations such as suboptimal targeting, production yield, efficacy, and dosage persist. To address these challenges, diverse engineering approaches are implemented to refine EVs for clinical applications. These engineering strategies focus on modifying the parental cells or augmenting the isolated EVs directly.

### Parental cell modification

**Fig. 2:**
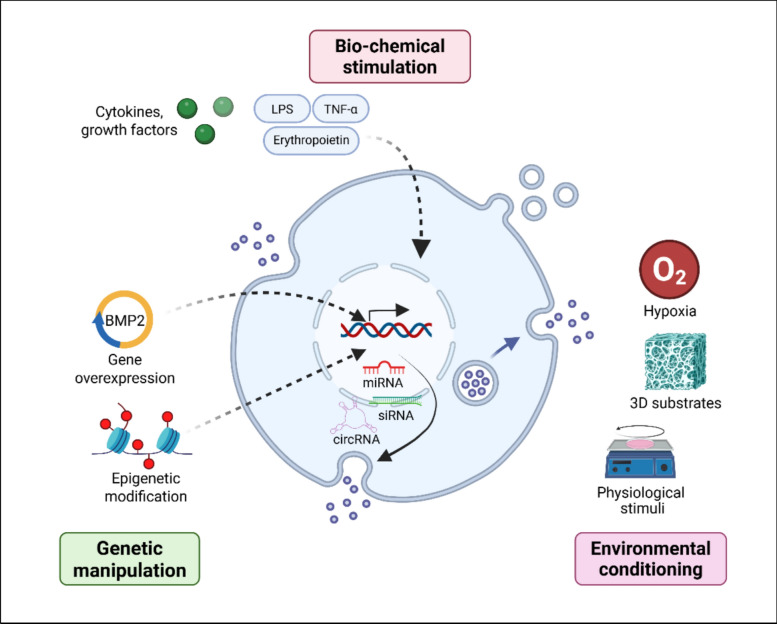
Parental cell EV engineering strategies. this figure summarizes various strategies for functionally engineering EVs through parental cell modification: biochemical stimulation modifies EV cargo via parent cell treatment (e.g., cytokines, growth factors, drugs). genetic manipulation through gene overexpression or epigenetic editing alters parent cell genes to influence EV content (e.g., miRNAs, circRNAs etc). environmental conditioning uses external cues (e.g., hypoxia, 3D substrates, physical stimulation) to modulate EV quantity and potency

Cells are highly receptive to physiological cues, augmenting important biological processes (i.e. proliferation, differentiation, apoptosis etc). Due to their plastic nature, this provides an opportunity to modify parental cell phenotype, stimulating the production of EVs with enhanced therapeutic potency. Here will we introduce several state-of-the-art bioengineering approaches explored in the literature, including biochemical stimulation, genetic manipulation, and environmental conditioning (Fig 2).

#### Biochemical stimulation

Biochemical stimulation using exogenous bioactive factors (i.e. drugs, growth factors, cytokines) is a well-establish strategy to improve EV functionality through modifying parental cell behaviour [31].

**Fig. 3:**
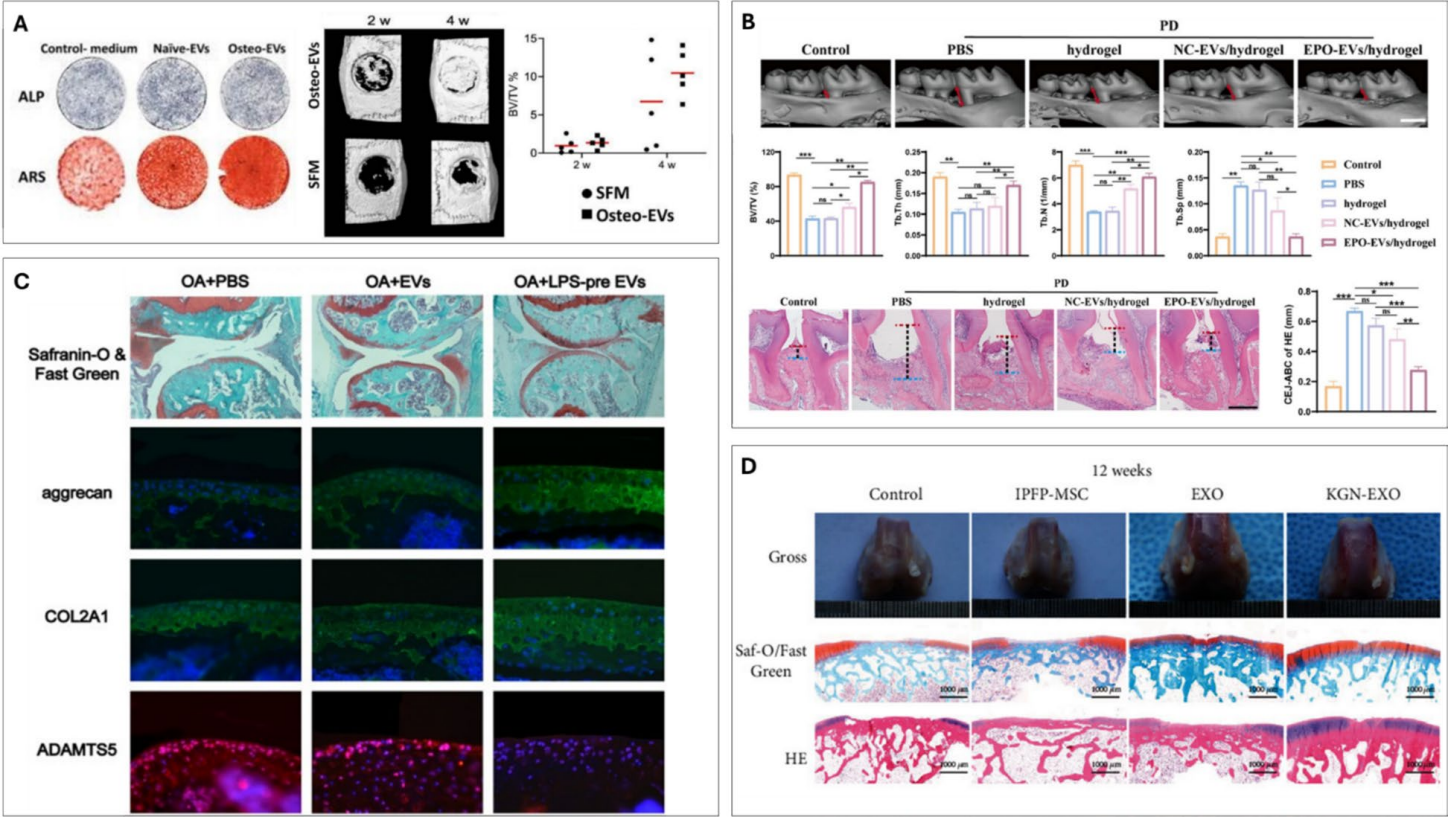
Biochemical stimulation to engineer EVs for orthopaedic applications. (**A**) alkaline phosphatase and alizarin red staining of Native-EVs and Osteo-EVs treatment on hMSCs. Representative µCT images and quantification of serum free medium (SFM) and Osteo-EVs treatment after 2 and 4 weeks in a rat calvarial defect model. Adapted from [114] under the creative commons license, 2025. (**B**) representative µCT images and quantitative analysis of maxillary samples. H&E staining and quantification analysis of the distance from CEJ to ABC of the periodontium in each group. Adapted from [119] under the creative commons license, 2025. (**C**) safranin-O/fast green and immunofluorescence staining of each group of mouse knee section. adapted from [120] under the creative commons license, 2021. (**D**) the gross appearance, safranin-O/fast green and H&E staining of *in vivo* cartilage repair at 12 weeks after surgery. adapted from [122] under the creative commons license, 2021

For bone applications, a common strategy to enhance the osteogenic potential of MSC EVs involves stimulating MSCs osteogenic differentiation [114, 115]. For example, Al-Sharabi et al. revealed that the differentiation status of MSCs significantly influences the osteogenic potential of their EVs [114]. Osteogenically pre-differentiated MSCs produced Osteo-EVs enriched with bone-related proteins, which compared to Naïve-EVs, exhibited superior *in vitro* osteoinductive capabilities (Fig 3A). Crucially, Osteo-EVs significantly enhanced bone regeneration in a rat calvarial defect model. Additionally, preconditioning MSC cultures with exogenous biomolecules, such as cytokines or drugs, has been explored [116]. Lu et al. used tumour necrosis factor-alpha (TNF-α) to mimic the inflammatory phase post injury, demonstrating that EVs from these preconditioned MSCs exhibited enhanced capacity to promote proliferation and differentiation in human osteoblastic cells *in vitro* [117]. Nakao et al. reported EVs procured from TNF-α-treated human gingiva-derived MSCs, promoted M2 macrophage polarisation, and reduced periodontal bone loss in a ligature-induced periodontitis model in mice [118]. Lui. et al. investigated the influence of erythropoietin (EPO) on modifying macrophage-derived EV potency for bone regeneration [119] (Fig. 3B). The authors showed that EPO-EVs rescued inflamed BMSC osteogenic fate by delivering miR-5107-5p, which targets and inhibits EGFR, thereby modulating the EGFR/RhoA axis to counteract inflammation-induced osteogenic suppression. Staubli et al. introduced a developmentally inspired preconditioning strategy to engineer hypertrophic cartilage microtissue capable of generating matrix vesicles with enhanced osteoinductive potency [66]. By mimicking the endochondral ossification process, the authors demonstrated that preconditioning MSCs toward a hypertrophic phenotype using TGF-β1 and BMP2 generated matrix vesicles enriched in pro-mineralization cues and signaling molecules, which significantly improved bone regeneration. This work highlights the potential of leveraging developmental preconditioning to bioengineer EVs with tailored regenerative functions.

For cartilage applications, Duan et al. investigated preconditioning of human synovial MSCs with lipopolysaccharide (LPS) to improve the therapeutic efficacy of EVs for OA treatment [120] (Fig 3C). The authors showed that LPS-EVs enhanced chondrocyte proliferation, inhibited apoptosis and ECM degradation compared to control EV treatment. The LPS-EVs were found to be enriched in Let-7b which inhibited the expression of the A disintegrin-like and metalloproteinase domain with thrombospondin-1 motifs 5 (ADAMTS5). Moreover, intraarticular injection of LPS-EVs prevents the development of OA within a destabilization of the medial meniscus-induced mouse model. The small bioactive molecule kartogenin (KGN) was used to pre-condition BMSCs as it has been reported to improve chondrogenic differentiation. EVs derived from these KGN pre-conditioned BMSCs increased cartilage-like matrix synthesis and limited degradation [121]. Similarly, Shao et al. demonstrated that EVs secreted from KGN pre-treated infrapatellar fat pad MSCs enhanced articular cartilage repair in rabbits [122] (Fig 3D). Li et al. reported a strategy leveraging curcumin-preconditioned BMSCs to generate therapeutic EVs (Cur-EVs) [123]. Their findings demonstrated that these Cur-EVs, when applied to IL-1β-stimulated primary human articular chondrocytes, upregulated the expression of miR-126-3p. This upregulation correlated with enhanced chondrocyte viability, reduced apoptosis, and decreased phosphorylation of key components within pro-inflammatory signalling cascades. The authors concluded that the elevated miR-126-3p mediated these protective effects by suppressing the MAPK, NF-κB, and PI3K/Akt pathways, which are critically involved in OA progression. These results provide compelling evidence for the anabolic potential of Cur-EVs in the context of OA. In another study, Lui et al. developed bioenergetic-active EV, rich in ATP, that enhance cartilage regeneration and homeostasis [124]. Succinate treatment activated the mitochondrial TCA cycle and elevated mitochondrial membrane potential, generating more endogenous ATP in BMSCs. Following succinate conditioning, Suc-EVs exhibited increased ATP content and higher levels of metabolites associated with energy metabolism, promoting BMSC chondrogenic differentiation via P2X7-PI3K-AKT and improving chondrocyte anabolism/mitochondrial homeostasis via P2X7-SIRT3. *In vivo*, Suc-EVs significantly improved cartilage repair and neocartilage maintenance in rabbits, highlighting a novel metabolic modulation strategy for tissue engineering. Taken together, these studies demonstrate the considerable potential of exploiting biochemical cues to engineer EVs for enhanced efficacy in regenerative orthopaedics.

#### Genetic and epigenetic modifications

**Fig. 4:**
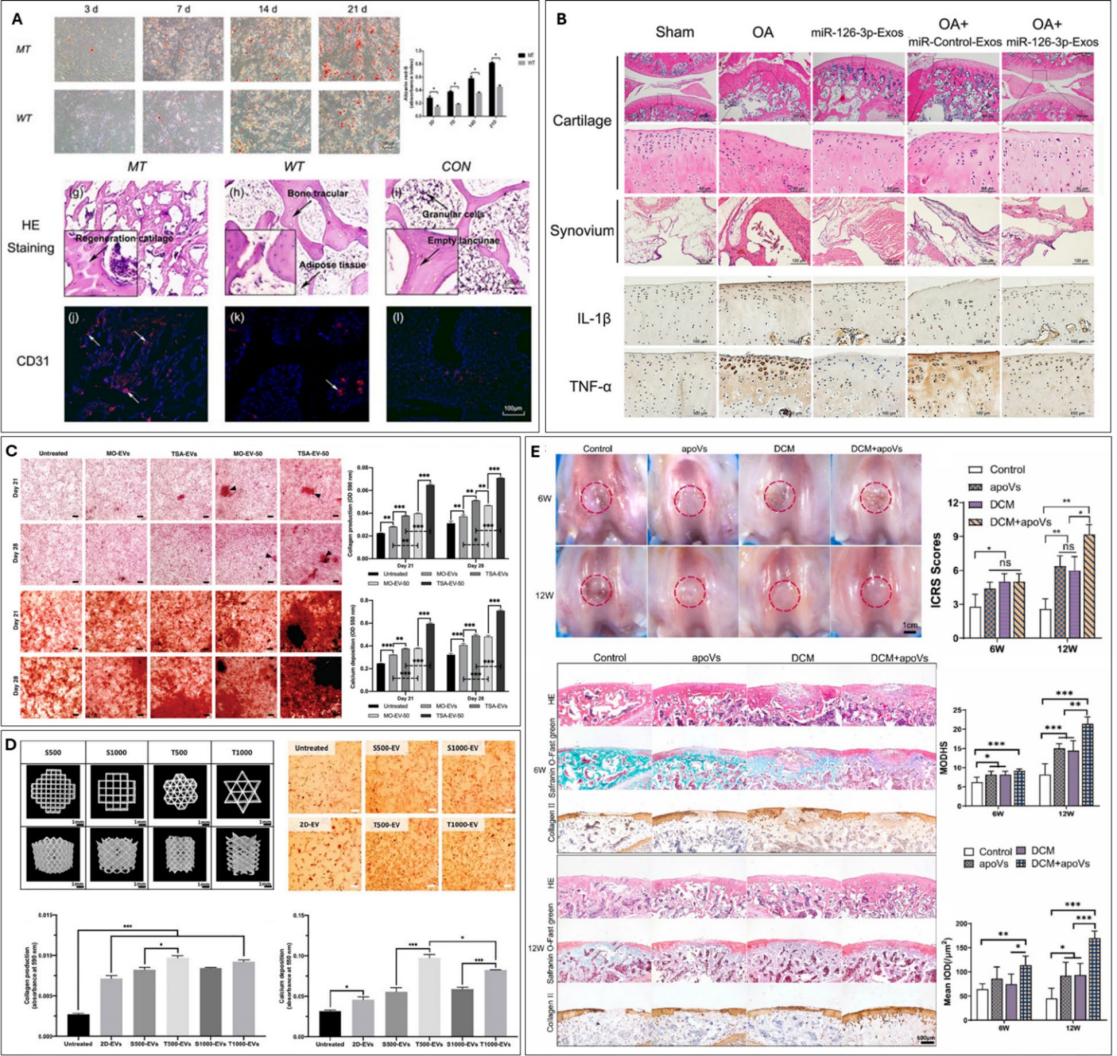
Genetic manipulation of EV parental cells through genetic (A, B) or epigenetic reprogramming (C, D, E) strategies. (**A**) alizarin red-S staining and quantification of BMSC-ExosMT and BMSC-ExosWT-treated BMSCs. H&E staining and CD31 immunohistochemistry of bone regeneration of femoral head necrosis sections at 6 weeks after treatment. adapted from [128] with permission from John Wiley and Sons, 2017. (**B**) H&E staining and immunohistochemical staining of rat articular cartilage and synovium treated with SFC-miRNA-126-3p-EVs. adapted from [132] under the creative commons license, 2021. (**C**) picrosirius red staining and quantification for collagen production of TSA EV treated hBMSCs. alizarin red staining and quantification for calcium deposition of TSA EV treated hbmscs. adapted from [35] under the creative commons license, 2021. (**D**) Representative µCT images of 3D printed titanium scaffolds. effect of scaffold-derived osteoblast EVs on hBMSC’s osteogenic via assessment of collagen production and mineralisation. adapted from [64] under the creative commons license, 2021. (**E**) representative microscopic observation and ICRS score for macroscopic assessment. Histological staining (H&E, safranin O/fast green, collagen II) of the osteochondral defect at 6 and 12 weeks. MODHS histological evaluations and quantitative analysis of COL2 of repaired cartilage. adapted from [136] with permission from Elsevier, 2024

One of the most common cell augmentation strategies include genetic modification. This process involves inserting a gene-of-interest into a cell, which is then overexpressed resulting in modifications in the cell’s secretome [125]. Lai et al*.* transfected BMSCs by complexing DP7-C, a novel cholesterol-modified peptide, with miR-26a to augment the osteogenic potency of the BMSC EVs [126]. The transfection led to a 300 times increase in EV production, and the vesicles were found to be enriched with miR-26a. These engineered EVs enhanced the migration, proliferation and osteogenic differentiation of recipient BMSCs as well as inducing bone regeneration *in vivo* in a periodontitis model in mice. Zhang et al. reported that miR-29b-3p was significantly down regulated in BMSC-derived EVs from osteoporotic patients when compared to non-osteoporotic patients [127]. The authors overexpressed miR-29b-3p within BMSCs, and the resulting EVs improved osteogenesis via blocking Suppressor of Cytokine Signalling 1/Nuclear Factor-κB Pathway by inhibiting the histone demethylase activity of lysine demethylase 5 A (KDM5A). Li et al. engineered BMSCs to express a mutant HIF-1α that remains stable under normal oxygen conditions [128]. EVs derived from these modified cells significantly enhanced the osteogenesis of BMSCs and promoted tube formation in human umbilical vein endothelial cells (HUVECs) *in vitro*. Crucially, in a rabbit model of early-stage avascular necrosis of the femoral head, these EVs markedly improved both angiogenesis and bone regeneration (Fig 4A). Huang et al. overexpressed BMP2 in MSCs to improve EVs osteoinductive capacity [129]. Interestingly, despite successful BMP2 overexpression, the authors showed the secreted EVs did not contain the BMP2 protein. These EVs were enriched with specific miRNAs that effectively potentiate the BMP2 signalling pathway in recipient MSCs. The researchers observed superior regeneration of rat calvaria defects compared to unmodified EVs, highlighting an indirect mechanism, by which genetically engineered MSCs can confer therapeutic benefits through their EVs.

For cartilage applications, Mao et al. demonstrated that EVs derived from chondrocytes overexpressing miR-95-5p enhance the chondrogenic differentiation of MSCs and stimulate the expression of cartilage matrix by chondrocytes. The overexpression of miR-95-5p also suppresses the expression of histone deacetylase 2/8 (HDAC2/8), which is known to be upregulated in OA [130]. In a similar study, He et al. revealed that EVs from miRNA-210-overexpressing BMSCs protected chondrocytes from LPS-induced injury [131]. These engineered EVs notably boost chondrocyte proliferation and reduce apoptosis by attenuating the NF-κB pathway. Zhou et al. identified the critical role for synovial fluid-derived EVs miRNA-126-3p, found downregulated in OA, in chondrocyte health [132] (Fig 4B). Reintroducing this miRNA via engineered synovial fibroblast EVs (SFC-EVs) promoted chondrocyte proliferation/migration, suppressed apoptosis/inflammation *in vitro*, and remarkably inhibited osteophyte formation and cartilage degeneration in a rat OA model, positioning SFC-miRNA-126-3p-EVs as a promising OA therapeutic. While genetic engineering of EV parental cells holds promise, challenges remain regarding transduction efficiency, high costs, and lengthy timelines [31, 133, 134]. Furthermore, safety considerations associated with genetically modified cells will likely necessitate more stringent clinical safety evaluations compared to EVs from non-genetically modified cells [135].

Epigenetic modifications are natural events occurring in the nucleus that alter transcriptional potential. An example of this includes the post-translational modifications on histones influencing chromatin structure, leading to changes in gene expression without modifying the DNA sequence [137, 138]. Thus, epigenetic editing offers a potentially reversible, safer, and more cost-effective alternative to genetic modification. Several studies have reported that epigenetic reprogramming enhances the efficacy of stem cell therapies for bone tissue engineering *in vitro* and *in vivo* [139–141]. Furthermore, cells are known to secrete EVs that contain molecules that mediate epigenetic reprogramming in recipient cells, including non-coding RNAs (i.e. circular RNAs (circRNAs), miRNAs, and long noncoding RNAs (lncRNAs)) and protein-based epigenetic regulators [142, 143]. Man et al. induced osteoblast hyperacetylation with the histone deacetylase inhibitor Trichostatin A, significantly enhancing EVs’ therapeutic efficacy by promoting recipient hBMSC recruitment, histone acetylation, and mineralization compared with unmodified osteoblast EVs *in vitro* [35] (Fig 4C). These ‘Epi-EVs’ were enriched with pro-osteogenic miRNAs and proteins involved in transcriptional regulation and ECM mineralization, synergistically promoting regeneration. Environmental stimulation of EV-producing cells can also be enhanced through epigenetic reprogramming. For example, treating hBMSCs with the hypoxia-mimetic agent deferoxamine (DFO) and the DNA methyltransferase inhibitor 5-azacytidine (AZT) resulted in EVs (AZT/DFO-EVs) that significantly increased recipient hBMSC mineralization, and augmented pro-angiogenic cytokine release from HUVECs [144]. This highlights the potential of combining hypomethylation and hypoxia of MSCs to improve the therapeutic potency of EVs. Moreover, studies have shown that material-induced epigenetic reprogramming alters the potency of secreted EVs. For instance, titanium microcarriers enhanced osteoblast mineralization when they exhibited a triangular pore shape, by stimulating histone hyperacetylation, compared with cells cultured on square-pore microcarriers [64]. Notably, EVs derived from these osteoblasts grown in triangular pore microcarriers improved hBMSC osteogenic differentiation (Fig 4D). These findings demonstrate the capacity of biophysical cues to influence EV potency through epigenetic modifications.

CircRNAs play a pivotal role in the epigenetic regulation of gene expression at transcriptional and post-transcriptional levels [145]. This study engineered synovium MSC-derived EVs to deliver the sleep-associated circRNA, circRNA3503, for OA therapy. Identifying circRNA3503 as upregulated and chondroprotective during melatonin-induced chondrocyte "sleep," the researchers generated EVs overexpressing this circRNA (circRNA3503-OE-EVs). *In vitro* and *in vivo* OA models showed that circRNA3503-OE-EVs mitigated OA progression by reducing inflammation-induced chondrocyte death and restoring matrix balance, highlighting the potential of epigenetically enhanced EVs for OA regeneration [146]. In a similar study, Mao et al. using MSC EVs enriched with circRNA0001236, demonstrated enhanced chondrogenesis and suppressed cartilage degradation *in vitro* and *in vivo*. Mechanistically, the study revealed that the EV-associated circRNA0001236 acts as a "sponge" for miR-3677-3p, thereby upregulating the expression of its target gene, Sox9, a master regulator of chondrogenesis [147]. Tian et al. reported the role of EV-associated epigenetic regulators in cartilage regeneration [136]. The authors reported that hUC-MSCs delivered within cartilage defects underwent substantial apoptosis, resulting in the subsequent release of apoptotic EVs. It was reported that EV-enriched miR-100-5p promoted M2 polarisation via the MAPK/ERK pathways. Moreover, this study highlighted the role of another EV-associated epigenetic marker, let-7i-5p on enhancing MSCs chondrogenic differentiation by activating the eEF2K/p38 MAPK axis. These mechanisms synergistically support cartilage regeneration and enhance the therapeutic potential of MSC-derived EVs (Fig 4E). These studies demonstrate the epigenetic manipulation of EV cargo to modulate the recipient cell’s gene expression and promote cartilage repair, representing a novel strategy in EV-based regenerative medicine. Taken together, these findings demonstrate the potential of gene manipulation through genetic or epigenetic reprogramming to augment EVs for orthopaedic regeneration.

#### Environmental conditioning

Exploiting environmental cues has been employed to mimic the physiological conditions and consequently cell behaviours observed *in vivo*. This has led to extensive investigations into exploiting environmental stimulation to improve EV therapeutic efficacy.

##### Hypoxia

Recognizing the inherent hypoxic environment of the bone marrow niche, several studies have reported the importance of hypoxic conditions in promoting the lineage-specific differentiation of progenitor cells and stimulating both bone and cartilage repair [148, 149]. Thus, researchers have explored exploiting hypoxic conditioning to augment EV function. For instance, Li et al. demonstrated that hypoxia preconditioning of ADSCs (1% O_2_ for 24 h) enhances the therapeutic potential of their derived EVs (hypo-ADSC-EVs) for osteoporotic fracture repair when delivered via a gelatin methacryloyl (GelMA) hydrogel [150]. These hypo-ADSC-EVs, enriched with miR-21-5p, promote Type H (bone specific) angiogenesis and bone regeneration by targeting SPRY1 and activating the PI3K/AKT pathway in endothelial cells. This highlights a combinatorial strategy to improve fracture healing through optimized EV delivery and pro-angiogenic signalling. Researchers have also investigated chemically inducing hypoxia using hypoxia mimetic agents. Man et al. exploited the hypoxia mimetic agent DFO (10 μM for 24 h) to induced hypoxia within MSCs stimulating HIF-1α expression. The authors showed that EVs procured from DFO-treated MSCs, exhibited enhanced osteogenic and angiogenic potency [144]. Similarly, Liang et al. treated MSCs with dimethyloxaloylglycine (DMOG, 1000 μM for 48 h) and reported that the DMOG-treated MSC EVs promoted HUVEC angiogenesis *in vitro* and calvarial bone defect healing in rats [151].

**Fig. 5:**
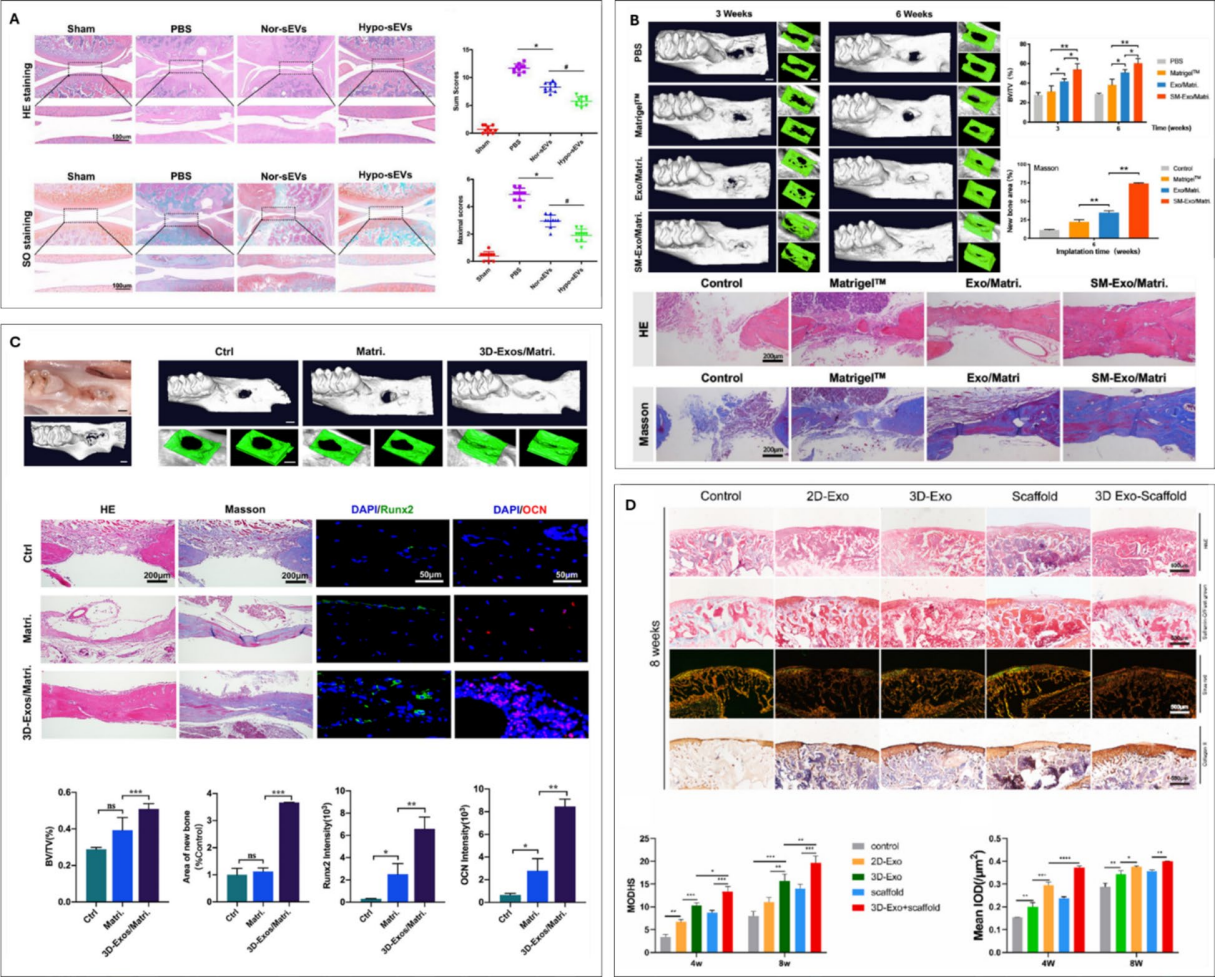
Environmental conditioning to improve EV potency for regenerative orthopaedics. (**A**) hypo-sEVs in the destabilization of the medial meniscus (DMM) model alleviated OA. representative images of H&E and safranin O/fast green staining of knee joint sections. evaluation of cartilage destruction using the OARSI system. adapted from [152] with permission from Elsevier, 2021. (**B**) representative µCT analysis of new bone formation in rat alveolar defects treated with PDLSC exosomes at 3 and 6 weeks of healing. Histological analysis (H&E, Masson’s trichrome staining) of exosome-treated bone defects. Adapted from [155] with permission from Elsevier, 2021. (**C**) treatment with 3D-Exos promotes developmental in situ osteogenesis. representative photos and µCT images of saline (control), Matrigel™, or 3D-Exos at six weeks of healing. Histological staining (H&E, Masson’s trichrome) and immunofluorescence staining of Runx2 and OCN. The BV/TV values, new bone area, and semi-quantification of Runx2 and OCN intensity. Adapted from [156] with permission from Elsevier, 2022. (**D**) H&E, safranin-O, sirius red and collagen II staining of repaired tissue after 8 weeks. MODHS histological evaluations and quantitative analysis of Collagen II. Adapted from [157] with permission from Elsevier, 2023

Consistent with these observations, Rong et al. demonstrated that hypoxic preconditioning of BMSCs (1% O_2_ for 48 h) not only increased EV secretion but also endowed these vesicles with enhanced chondroprotective effects [152]. Specifically, these EVs promoted chondrocyte migration and inhibited apoptosis *in vitro*, mediated by the miR-216a-5p/JAK2/STAT3 signalling pathway. Furthermore, in a rat OA model, these hypoxia-primed EVs attenuated cartilage degeneration and facilitated repair (Fig 5A). Supporting this line of evidence, Zhang et al. reported that EVs from hypoxia-preconditioned MSCs (5% O_2_ for 48 h) similarly enhanced chondrocyte proliferation and migration while suppressing apoptosis [153]. Their analysis of EV miRNA cargo implicated the miRNA-18-3P/JAK-STAT and miRNA-181c-5p/MAPK signalling pathways in mediating the beneficial effects of hypoxia. Similarly, Shen et al. demonstrated that hypoxia-preconditioned (3% O_2_ for 48 h) BMSC-derived EV significantly enhance chondrocyte proliferation, migration, and anabolism while reducing inflammation via the miR-205-5p/PTEN/AKT pathway [154]. The combination of EVs within a silk fibroin hydrogel and chondrocytes effectively promote cartilage defect regeneration, highlighting hypoxia preconditioning and hydrogel delivery as promising strategies for cartilage tissue engineering. Collectively, these studies underscore hypoxic preconditioning of cells as a promising and consistently effective strategy for generating EVs with augmented therapeutic capabilities for the treatment of orthopaedic damage.

##### Mechanical stimulation

Biophysical cues play a major role in the development, homeostasis and pathogenesis of several musculoskeletal diseases [158]. Due to their important role in regulating cell fate, researchers have explored the use of biomechanical stimulation to engineering pro-regenerative EVs.

Biomechanical stimulation has been established as a potent method to improve the osteogenic phenotype of cells, naturally leading to adoption as an EV engineering strategy. Yu et al. applied 20% magnetic-induced strain for 72 h on human periodontal ligament stem cells (hPDLSCs) within a collagen/Fe_3_O_4_ hydrogel [155]. The authors showed that EVs from mechanically stimulated cells exhibited significantly altered miRNA profile and accelerated alveolar bone defect repair in rats compared to EVs produced from static cultures (Fig 5B). Similarly, Morrell et al. found that mechanical stimulation, through axial tibia compression at anabolic loading levels (35 dynes/cm^2^ for two 10-min bouts of steady flow separated by a 15 min rest period, for 36 h), induces intracellular calcium oscillations in osteocytes (MLO-Y4 cells) modulating LAMP1 expression which in turn triggers the release of EVs. These mechanically induced EVs were shown to be enriched in key bone regulatory protein (LAMP1, RANKL, OPG, and sclerostin), suggesting that the mechanosensitivity of osteocytes, transduced through calcium signalling and EV release, plays a crucial role in skeletal adaptation to mechanical cues [78]. Wu et al. developed a force-controlled 3D mechanical stretching of BMSCs embedding within a GelMA/hyaluronic acid methacryloyl (HAMA) hydrogel sheet to augment EV production [159]. Using a specialized micro-stretching manipulator, researchers demonstrated that applying precise, consistent mechanical force (loading parameters: 10% strain, frequency of 1 Hz for 7 days) significantly increased BMSC EV secretion when compared to static controls. These mechanically stimulated EVs exhibited superior osteogenic differentiation induction capabilities *in vitro* and notably accelerated bone repair in a rat calvarial defect model.

In a recent study, Luo et al. explored the influence of hydrostatic pressure on EV production from MSC during chondrogenic culture [160]. The application of hydrostatic pressure (amplitude of 270 kPa at a frequency of 1 Hz, 1 h per day, 5 days per week) was found to enhance chondrogenic differentiation in both human embryonic stem cells (hESCs) embedded into fibrin gels and hBMSCs pellets, whilst also substantially increasing EV production yields from both cell types. Consistent with these findings, Yan et al. utilized a rotary cell culture system to achieve a greater than two-fold increase in EV yield from UC-MSCs (cultured on Cytodex 3 microcarriers, 15–48 rpm/min for 48 h) compared with static culture [161]. EVs derived from these mechanically stimulated cultures exhibited enrichment of LncRNA H19, which promoted chondrocyte proliferation and matrix synthesis while inhibiting apoptosis. Importantly, administration of these mechanically activated EVs enhanced cartilage repair in a rat cartilage defect model. Collectively, these studies underscore the significant impact of bio mechanical stimulation on the production of EVs with enhanced therapeutic properties for regenerative orthopaedics.

##### Biomaterial substrates

2D cell culture substrates are the most utilized for EV manufacture, however, these highly artificial surfaces do not mimic the native microenvironment *in vivo* [162]. The loss of native-like phenotype likely negatively impacts the therapeutic efficacy of produced EVs. There has been growing research demonstrating that EVs procured from 3D cultured cells exhibiting superior therapeutic outcomes when compared to those obtained from 2D culture [163].

For instance, Yu et al. investigated the advantages of 3D hydrogel systems for enhancing the production and therapeutic efficacy of EVs for bone regeneration [156]. The authors combined human periodontal ligament stem cells (hPDLSCs) in a collagen type I hydrogel, which demonstrated a 2.5-fold increase in EV yield when compared to 2D cultures. These 3D-derived EVs also significantly improved BMSC proliferation, migration, and osteogenic differentiation *in vitro*, linked to the upregulation of YAP signaling. Moreover, these EVs accelerated bone healing in a rat alveolar bone defect model when combined with Matrigel (Fig 5C). Recently, researchers have also explored the development of bone-mimetic 3D printed substrates to improve the production of osteoinductive EVs. For instance, nano-hydroxyapatite coated titanium scaffolds were 3D printed to exhibit a bone-mimetic architecture [64]. The authors showed that osteoblasts cultured on these 3D substrates exhibited accelerated mineralisation (>2.6-fold) and generated significantly enhanced EV yield (4.5-fold) when compared to cells cultured in 2D. Furthermore, the 3D scaffold-produced EVs enhanced recipient hBMSC osteogenesis when compared to EVs derived from 2D cultured osteoblasts. Gao et al. investigated the impact of 3D culture conditions on the pro-angiogenic potential of MSC EVs [164]. The researchers found that EVs secreted by MSCs cultured on a 3D printed hydroxyapatite scaffold exhibit enhanced pro-angiogenic activity compared to those from 2D cultures. Specifically, these 3D-derived EVs promoted endothelial cell proliferation, migration, and angiogenesis by activating the HMGB1/AKT signalling pathway, thereby promoting vascularization in tissue regeneration.

It is well known that chondrogenic progenitor cells require a 3D microenvironment to support chondrogenesis and prevent dedifferentiation, mimicking their microenvironment *in vivo* [165]. Yan et al. reported the EVs produced from 3D culture of UC-MSCs in a hollow-fiber bioreactor (fiber surface area was 80 cm^2^) stimulated chondrocyte proliferation and migration as well as inhibiting chondrocyte apoptosis *in vitro* when compared to EVs from UC-MSC in 2D culture [166]. Additionally, the authors showed that the 3D EVs were more effective in promoting cartilage repair within a rabbit cartilage defect model, by activating the TGF-β1 and Smad 2/3 signalling pathways. Similarly, researchers have explored enhancing the potency of EVs derived from hUC-MSCs for osteochondral repair by culturing the hUC-MSCs in a 3D porous scaffold [157]. They found that EVs produced under these 3D culture (porcine cartilage ECM 3D-printed scaffold) conditions demonstrated improved efficacy in promoting the regeneration of both bone and cartilage tissues when compared to EVs produced from hUC-MSCs on 2D ECM films (Fig 5D). Thus, replicating the 3D physiological microenvironment provides a useful strategy to re-engineer EVs with enhanced potency.

### Direct EV modifications

**Fig. 6:**
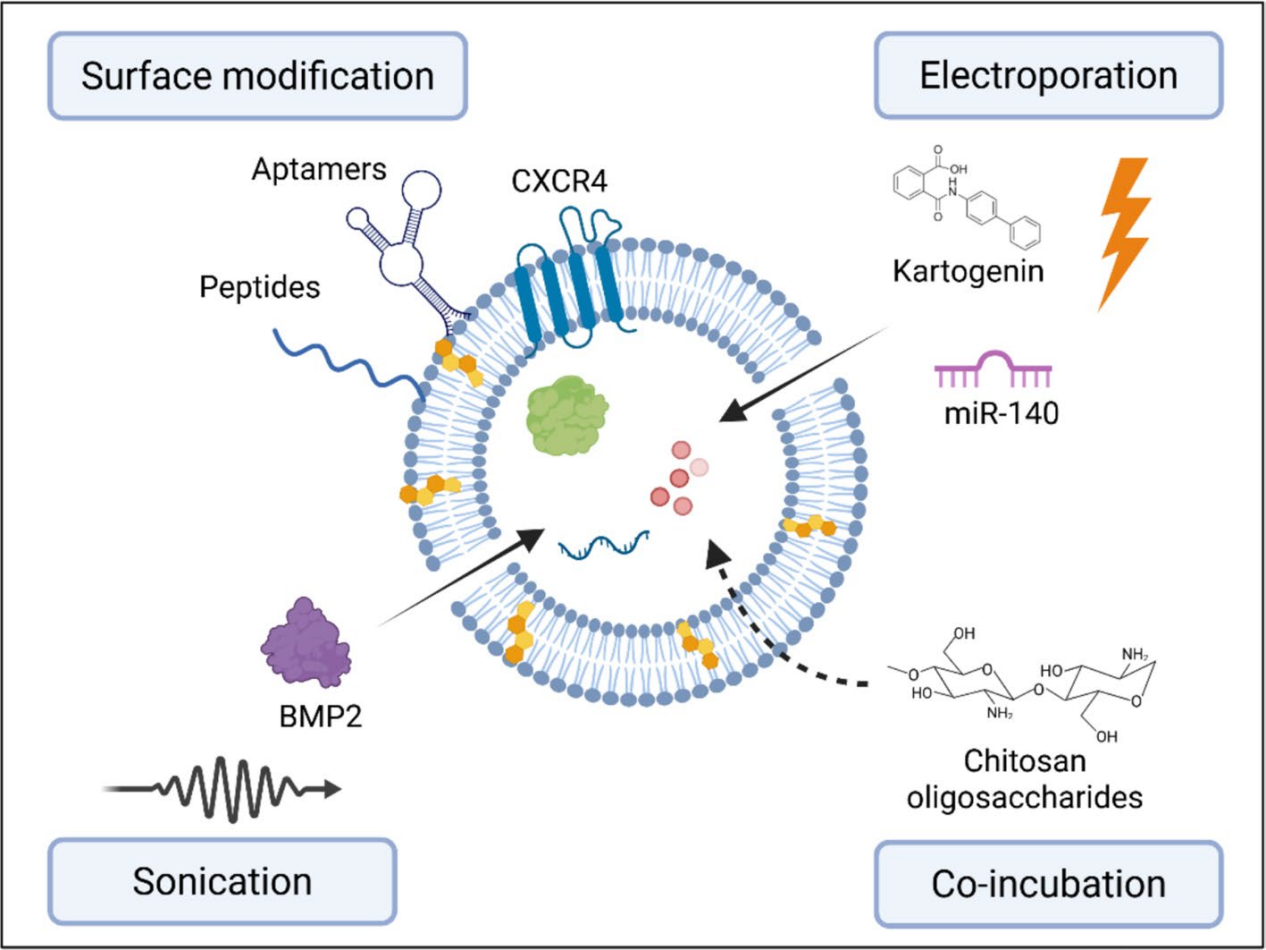
Direct loading methods to improve EV efficacy for regenerative orthopaedics. researchers have employed a variety of methods to endow EVs with enhanced potency, such as modifying their surface or by loading specific cargo to improve their therapeutic and targeting capabilities. Surface modification (top left) involves integrating or attaching molecules such as aptamers, peptides, or specific transmembrane receptors like CXCR4 to guide EVs to target cells or tissues. cargo loading (indicated by arrows into the EV lumen) can be achieved through several methods: electroporation (top right) utilizes electrical pulses to temporarily permeabilize the lipid membrane, allowing the encapsulation of diverse therapeutic agents like small molecules (e.g., kartogenin) or nucleic acids (e.g., miR-140). sonication (bottom left) employs ultrasound waves to facilitate the entry of larger molecules, such as proteins (e.g., BMP2). Co-incubation (bottom right) involves passive loading, often through simple incubation with molecules like chitosan oligosaccharides

There has been extensive research exploring the modification of native EVs post-isolation to further improve their therapeutic function [31, 167]. These include either physical or chemical modifications to augment EV functionality, such as loading EVs with specific cargo of interest and improving their biodistribution. Such engineering strategies may be relevant for customizing EVs as advanced drug delivery systems (Fig 6).

#### EV cargo loading

EVs possess unique characteristics like high biocompatibility, physiochemical stability and low immunogenicity, which make them an ideal drug delivery system for several clinical applications. Loading of cargo post-isolation can occur using several methods, including electroporation, sonication, freeze-thaw cycle, or extrusion [167]. The choice of loading technique and the specific conditions employed are determined by the nature and physicochemical properties of the cargo [31]. For example, hydrophilic molecules and nucleic acids are commonly incorporated through electroporation or sonication, which transiently permeabilize EV membranes, whereas hydrophobic drugs or small molecules are efficiently loaded via incubation or passive diffusion methods. Thus, the selection of loading approach must be tailored to the cargo type to achieve optimal encapsulation efficiency and EV stability.

**Fig. 7:**
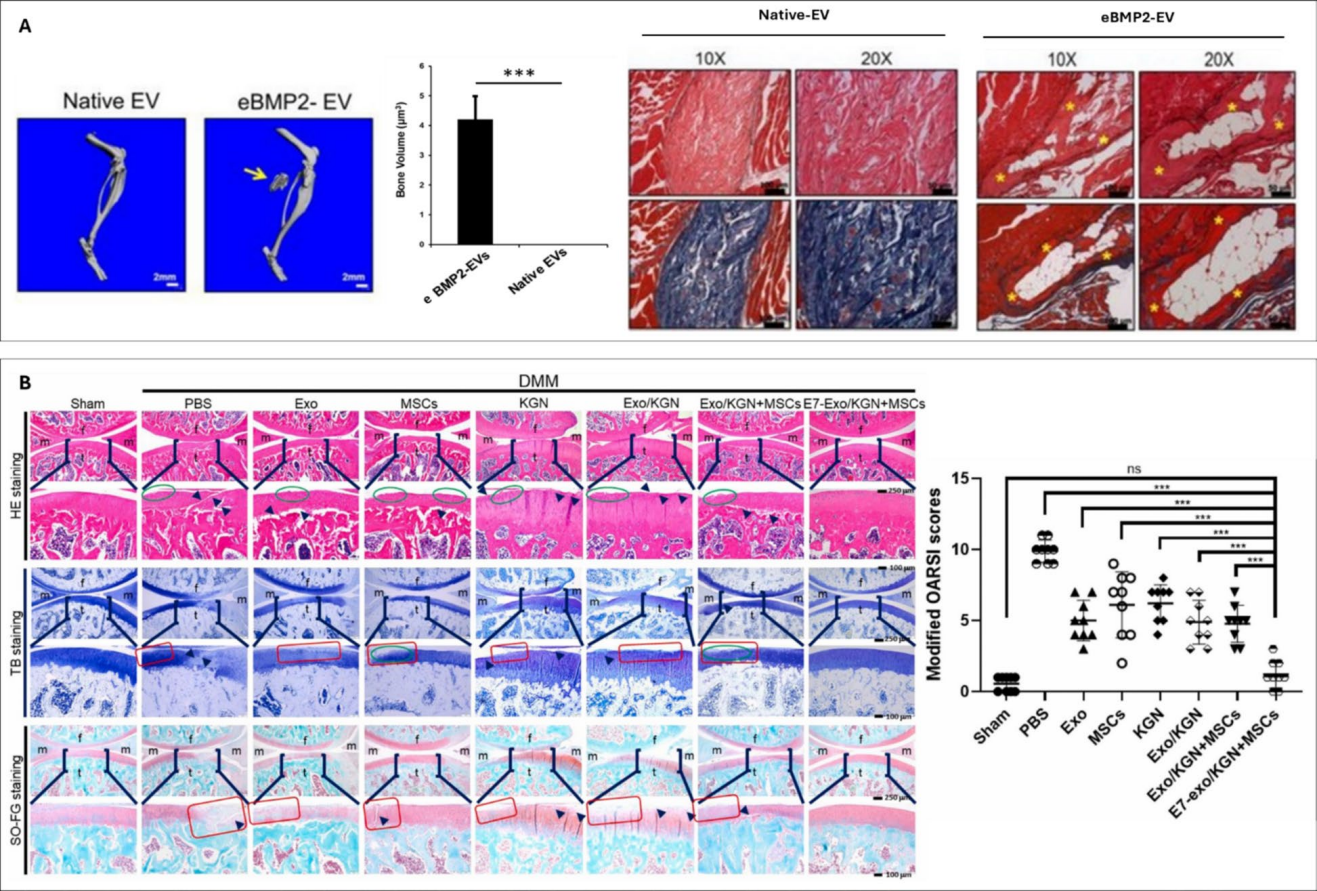
EV cargo loading for regenerative orthopaedics. (**A**) µCT images and quantification of heterotopic ossification with eBMP2-EVs constructs in murine muscle pocket model. representative H&E and Masson’s trichrome staining of native EVs and BMP2‐EVs implants (*indicates bone tissue). adapted from [170] under the creative commons license, 2021. (**B**) H&E, toluidine blue, safranin O/fast green staining. green circles indicate the coarse cartilage surface. black arrows indicate fractures of holes in the tissues. histological scores of the articular cartilage using the OARSI grading system. adapted from [173] with permission from Elsevier, 2021

Hu et al. fused liposomes carrying antagomir-188 with CXCR4-positive EVs to produce hybrid nanoparticles. The authors showed that these CXCR4-expressing nanoparticles specifically accumulated in the bone marrow, promoting BMSCs osteogenesis and alleviating age-related bone loss in mice [168]. In another study, Mi et al. loaded miR-26a-5p into endothelial cell-derived EVs using the CD9-HuR fusion protein [169]. These miR-26a-5p-loaded EVs were incorporated into a hyaluronic acid-based hydrogel for local delivery to the fracture site. The authors observed improved fracture healing in a femoral fracture model in mice. Yerneni et at. harnessed sonication or electroporation to load BMP2 into EVs derived from the murine J774A.1 monocytic cell line [171] (Fig 7A). The authors showed that sonication resulted in a 3-fold higher loading efficiency when compared to electroporation. The BMP2-loaded EVs stimulated *in vitro* osteogenesis of treated C2C12 and MC3T3 cells. The BMP-EVs were combined with a collagen‐rich acellular dermal matrix and implanted in a murine muscle pocket model. After 4 weeks implantation, there was evidence of ectopic ossification observed in the BMP-EV groups, whilst the native EV group displayed no bone formation.

Several studies have explored loading of therapeutic cargo into EVs to improve their efficacy for cartilage regeneration. For instance, Li et al. demonstrated that rat ADSC EVs, when co-incubated with chitosan oligosaccharides, promoted chondrocyte viability and migration, leading to improved cartilage repair and OA alleviation in rats [171]. Similarly, Liang et al. employed electroporation to load miR-140 into chondrocyte-derived EVs, showing that these targeted EVs effectively delivered miR-140 and attenuated OA in rat models [172]. The delivery of small bioactive molecules, such as KGN, also holds promise for cartilage regeneration. However, the clinical applicability of KGN is limited by its low water solubility. To address this, Xu et al. obtained EVs from synovial fluid MSCs and used electroporation to incorporate KGN into these vesicles [173]. Their findings showed that KGN-loaded EVs induced a greater degree of chondrogenic differentiation in synovial fluid MSCs compared to KGN alone. Furthermore, intra-articular injection of KGN-loaded EVs enhanced cartilage repair in a rat OA model when compared to KGN alone (Fig 7B).

While these studies highlight the potential of EV-loading strategies in regenerative orthopaedics, a delicate balance exists between preserving the native composition of EVs and maximizing cargo-loading efficiency. Consequently, the synergistic delivery of both native and exogenous cargo may offer the most effective approach to promote bone and cartilage repair.

#### Surface modification

To improve the targeting and drug-delivery efficiency of EVs, researchers have explored approaches to modify the EV membrane, such as by using aptamers and peptides.

**Fig. 8:**
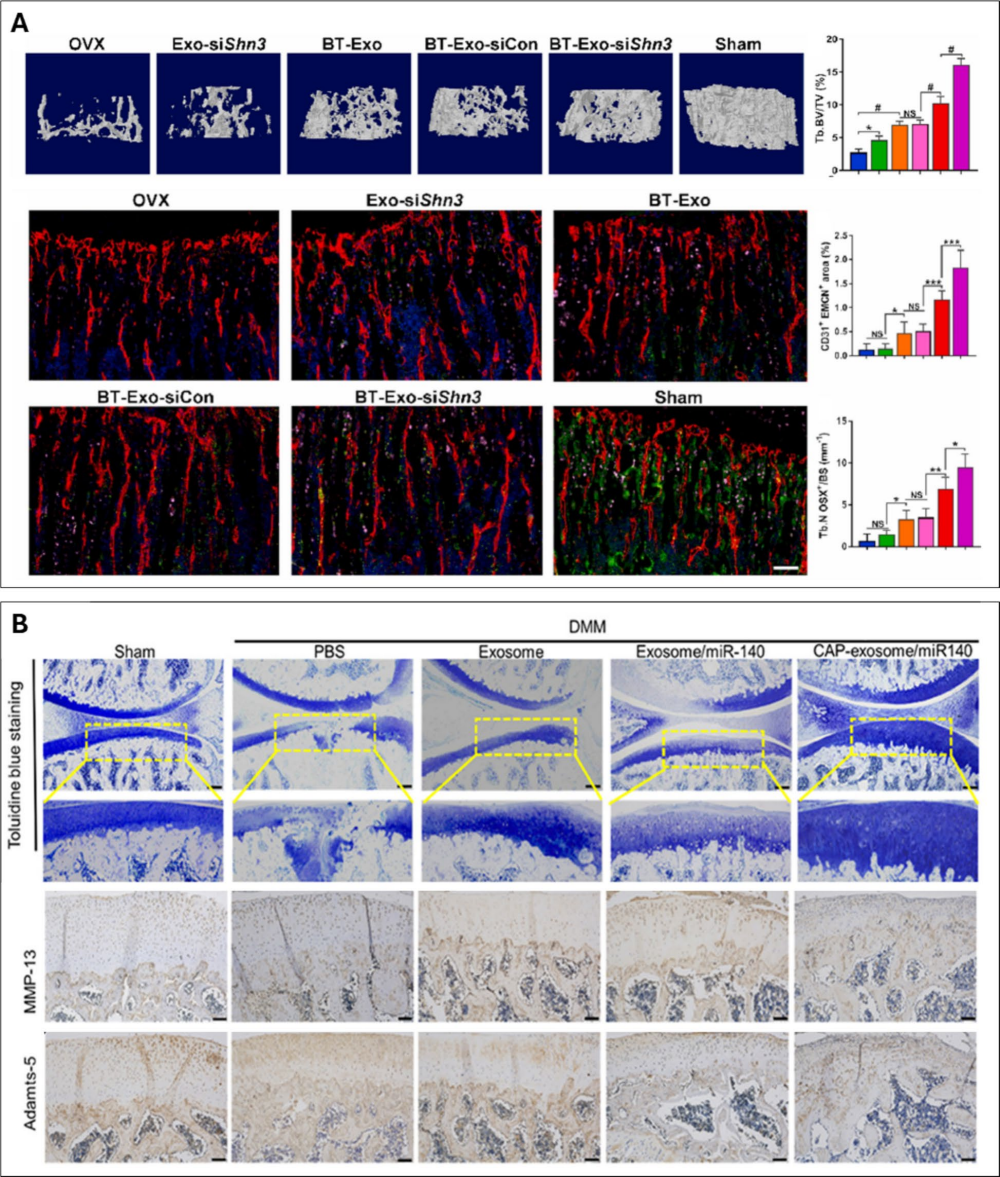
EV surface modification approaches to improve therapeutic efficacy. **(A**) representative µCT images and quantitative analysis showing trabeculae microarchitecture of distal femurs. immunofluorescent images of type H vessels, EMCN (red), CD31 (green), OSX (pink) and nuclei, DAPI (blue). quantitative analysis of type H vessels and osteoprogenitors. adapted from [178] under the creative commons license, 2022. (**B**) histological (toluidine blue) and immunohistochemical staining (MMP-13 and adamts-5) of the cartilage of DMM rats treated with CAP-exosome/miR-140 or exosome/miR-140. adapted from [172] with permission from American chemical society, 2020

Aptamers are short nucleic acid chains of RNA or single-stranded DNA, which have been increasingly employed to augment the targeting capacities of EVs [174]. It has been reported that the conjugation of MSC-specific aptamers to MSC EVs enhanced targeting in bone tissues, whilst reducing off-target accumulation (i.e. liver and lungs). Moreover, aptamer-functionalised EVs improved bone mass in an OVX (i.e. osteoporotic) mouse model, in addition to accelerating femoral fracture healing in mice [175]. In a similar study, Shou et al. created M2 macrophage-derived EVs functionalised with 3WJ RNA nanoparticles, displaying a BMSC-targeting aptamer for targeted bone fracture healing [176]. The study demonstrated that following the systemic administration of these EVs in mice, significantly accelerated bone defect repair was observed in a preclinical fracture model. Peptides, short proteins chains (<100 amino acids), have also shown great potential to promote tissue regeneration via the functionalisation of biomaterials [177]. Cui et al. developed a bone-targeted EV platform to deliver small interfering RNA (siRNA) for the treatment of osteoporosis [178]. The researchers manufactured EVs, secreted by MSCs derived from iPSCs. These were then modified by conjugation with the bone-targeting peptide DSPE-PEG-Mal-Cys-SDSSD and loaded with siShn3 via electroporation. The constructed bone-targeting EVs were able to specifically deliver siShn3 to osteoblasts, enhancing osteogenic differentiation and inhibiting osteoclast formation *in vitro,* and prevented OVX-induced bone loss in mice (Fig 8A)*.* To treat osteoporosis, Liu et al. obtained EVs from *Lactobacillus rhamnosus* bacterial cultures and anchored bone targeting peptides at the EV surface to deliver miRNAs to the bone microenvironment [179]. The engineered EVs exhibited bone tissue trophism, which was validated in a mouse model with bio-photonic imaging. Following intravenous injection into mice weekly for 8 weeks, acute and systemic toxicity was assessed through histopathological analysis of major organs. The authors showed no significant pathological changes in major organs compared to the PBS group, indicating the EVs were well-tolerated. The engineered EVs increased BMSC osteogenic differentiation, while inhibiting osteoclastogenesis of Raw264.7 cells *in vitro,* underscoring their potential as an osteoporotic treatment.

In the context of cartilage repair, altering EV surface charge has also been investigated as a strategy to improve cartilage penetration, overcoming the inhibitory effects of the negatively charged cartilage matrix. Feng et al. modified EVs with ε-polylysine-polyethylene-distearyl phosphatidylethanolamine to generate positively charged MSC-EVs, which exhibited enhanced cartilage matrix penetration [180]. Xu et al. used plasmid transfection of donor cells to enrich E7 peptide on the EV surface, thereby improving targeting to synovial fluid-derived MSCs and enhancing OA treatment efficacy [173]. Similarly, Liang et al. successfully generated chondrocyte-affinity peptide (CAP)-EVs by fusing the CAP with glycoprotein 2b, a membrane protein associated with lysosomes, on the surface of EVs [172]. In a rat model, the CAP-EVs transported miR-140 to the innermost layers of the cartilage by penetrating the thick perichondrium. This inhibited the proteases responsible for cartilage degradation and alleviated OA progression (Fig 8B). Taken together, the presented EV bioengineering approaches offer tremendous potential to maximize the therapeutic efficacy of EV-based therapies for orthopaedic regeneration.

## EV-mimetic systems

**Fig. 9:**
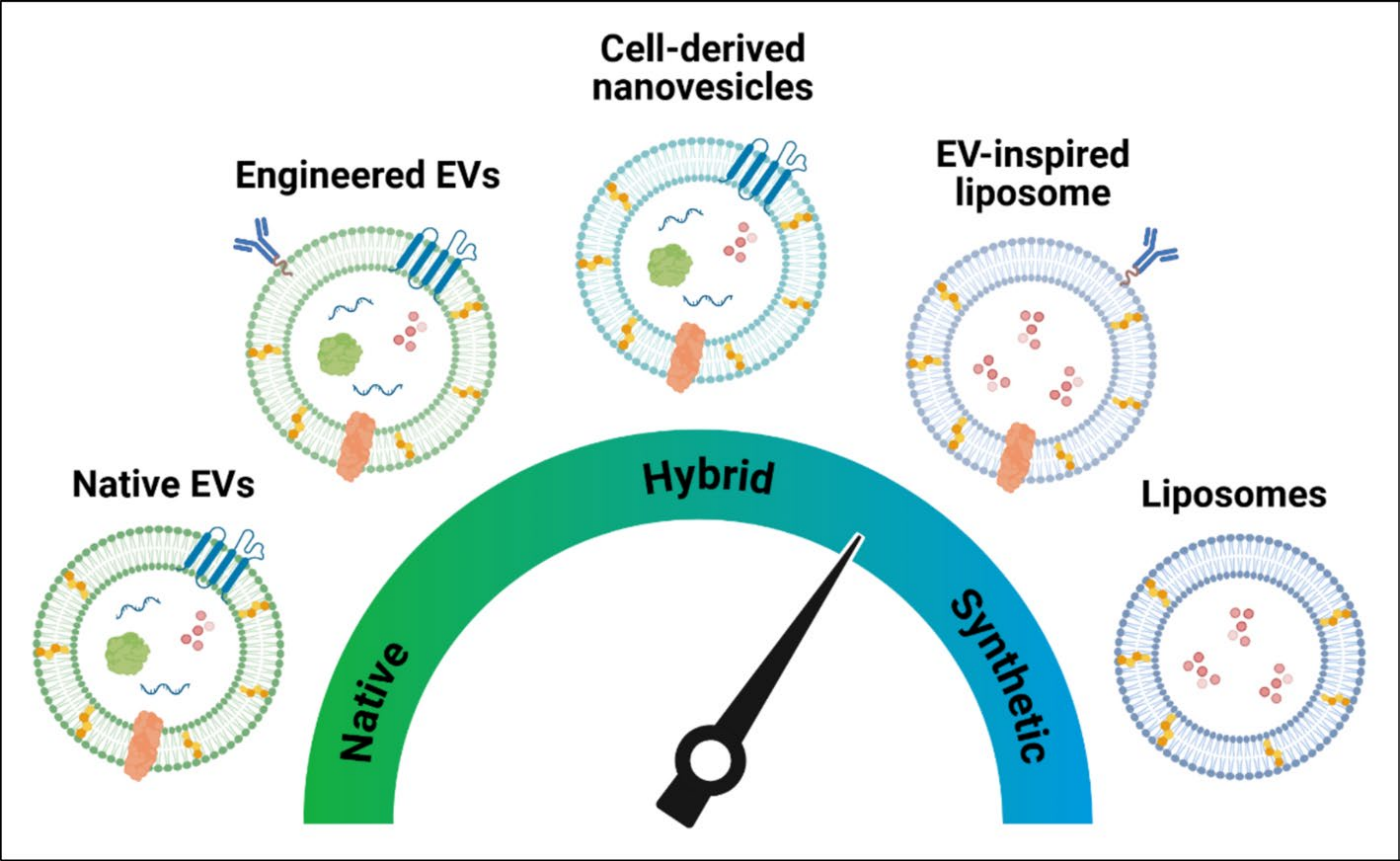
Overview of the EV-mimetic systems with varying degrees of complexity and biomimicry. this figure illustrates a spectrum of nanovesicle types, ranging from native to fully synthetic, with hybrid approaches bridging the two extremes. naïve or native EVs represent naturally occurring, minimally manipulated vesicles which can then be further engineered to improve therapeutic or targeting capabilities. examples of hybrid vesicles include cell-derived nanovesicles and hybrid EVs (fusogenic liposomes). EV-inspired liposomes are synthetic liposomes, designed to mimic certain characteristics of EVs. finally, liposomes represent fully synthetic lipid-bilayer vesicles

The development of EV-mimetics is an emergent research field, where these hybrid or fully synthetic nanoparticles aim to overcome key issues hindering the translation of EV-based therapeutics to the clinic (i.e. scalable manufacture, manufacturing costs, batch-to-batch variability etc). Understanding the mechanism, by which EVs induce their therapeutic function, has inspired researchers to develop EV-mimetics that mirror their function. EV-mimetic systems span a spectrum of complexity and biomimicry, ranging from minimally manipulated native EVs to fully synthetic lipid vesicles (Fig 9). Along this spectrum, cell-derived nanovesicles (CDNs) are generated by mechanically or chemically disrupting cells, retaining membrane proteins and cytosolic cargo while partially preserving the functionality of native EVs. Hybrid EVs, including fusogenic liposomes, are formed by combining natural EV components with synthetic nanocarriers to enhance stability, targeting, or cargo delivery. EV-inspired liposomes are fully synthetic vesicles designed to mimic key properties of EVs, such as size, lipid composition, and targeting capability, but do not contain native cellular biomolecules. At the far end of the spectrum, conventional liposomes are fully synthetic lipid-bilayer vesicles without direct EV-like bioactivity.

### Cell-derived nanovesicles

Cell-derived nanovesicles (CDNs) are lipid nanoparticles obtained through the physical disruption of whole cells, typically by mechanical extrusion or sonication [31, 181, 182]. The membranes then reform into nano-sized vesicles exhibiting similar physiochemical properties and contents of the parent cell [183]. This approach provides a rapid method of manufacturing cell-derived nanoparticles when compared to obtaining EVs [184]. Moreover, several studies have showcased the enhanced yields of nanoparticles generated through this approach compared to conventional EV isolation [185, 186]. Due to the reassembling of the plasma membrane during CDN manufacture, this provides the opportunity to additionally encapsulate therapeutic molecules within the nanoparticles, highlighting their potential as drug carriers [187, 188].

**Fig. 10:**
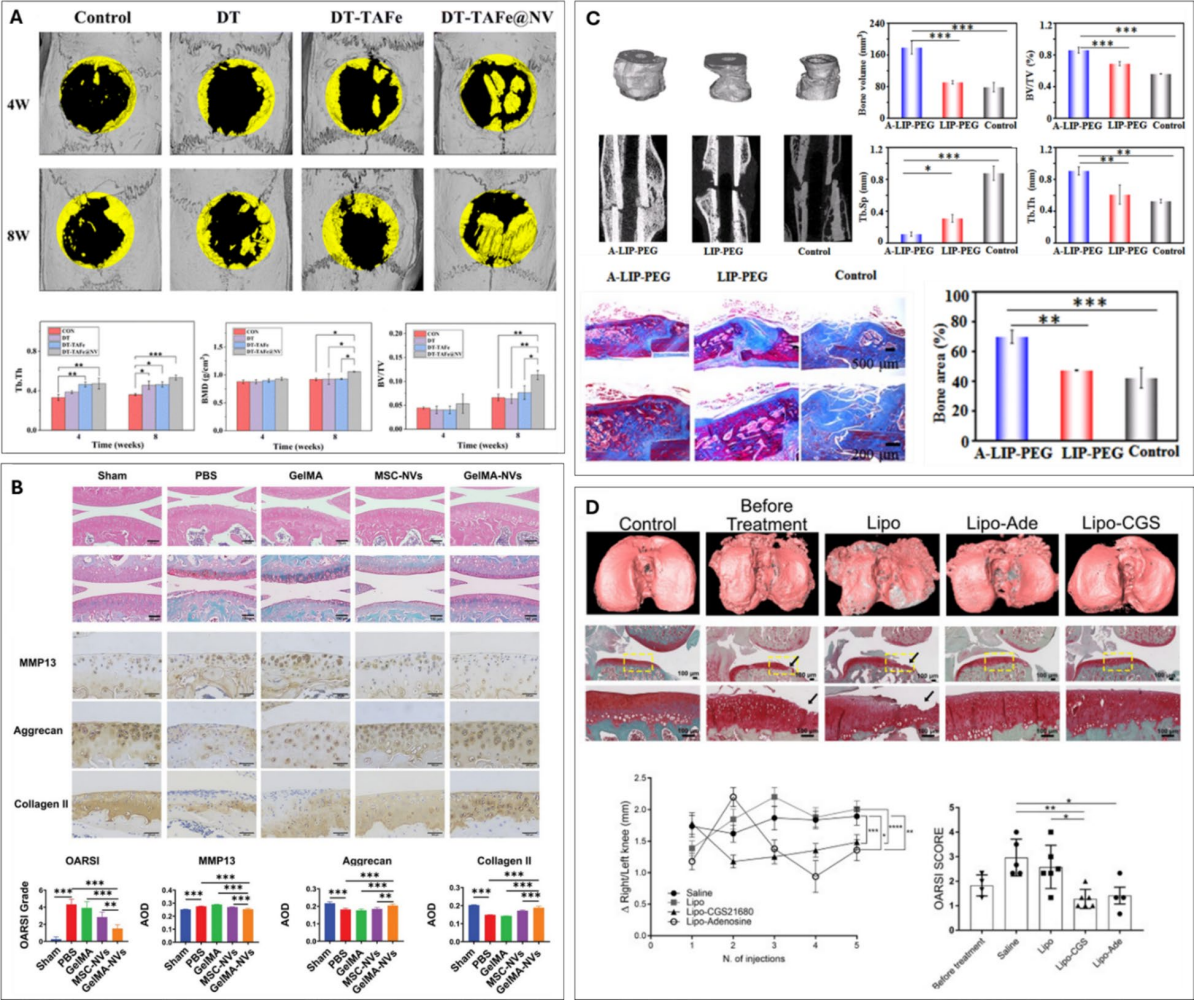
EV-mimetic systems for orthopaedic regeneration. (**A**) representative µCT images of rat cranial bone regeneration and quantitative analysis of new bone formation (yellow areas). adapted from [190] with permission from royal society of chemistry, 2025. (**B**) histological (safranin-O/fast green and H&E staining) and immunohistochemistry analysis (MMP-13, aggrecan, and collagen II) of tibial plateau sections. OARSI scoring of joint lesions. quantification of MMP-13, aggrecan, and collagen II staining. adapted from [185] with permission from John Wiley and Sons. (**C**) 3D reconstructed µCT images and quantification of bone morphology alterations in the fracture area after 8 weeks of treatment. masson’s trichrome staining of osteoporotic fracture areas and quantification of new bone formation. adapted from [191] under the creative commons license, 2019. (**D**) representative µCT images (cartilage = pink, underlying bone = dark grey). safranin-O/fast green stained sections. swelling measurement of the knee joint before every injection compared to the healthy knee of each rat. OARSI scores of the knees of the rats. adapted from [192] under the creative commons license, 2020

In regenerative medicine, there is growing evidence, demonstrating the promise of CDNs as EV-mimetic systems. For bone repair, Ravi et al. generated CDNs from the human embryonic kidney 293 cell line via extrusion and they were loaded with the potent osteoinductive glucocorticoid drug dexamethasone [189]. The authors showed that the dexamethasone-loaded CDNs were able to stimulate the osteogenic differentiation of ADSCs *in vitro* to a greater degree compared to the dexamethasone-free CDNs. Karoichan et al. explored CDNs from MSCs as a scalable EV-mimetic for bone regeneration [186]. Their study revealed that MSC-CDNs yielded twice the number of nanoparticles compared to conventionally isolated MSC-EVs and enhanced *in vitro* osteogenic differentiation of MSCs. Proteomic analysis further indicated an increased enrichment of osteogenesis-related proteins within MSC-CDNs compared to MSC-EVs. *In vivo*, using a femoral osteotomy model in mice, MSC-CDN treatment accelerated fracture healing, evidenced by promoted callus mineralization and reduced osteoclast activity. However, this effect was not directly compared to MSC-EV treatment, and given the non-critical defect size, spontaneous regeneration was also observed in control groups. Ma et al. generated CDNs obtained from stem cells of the apical papilla (SCAPs) and encapsulated these within a metal-phenolic network coating on a decellularized tendon matrix [190]. These SCAPs-CDNs, rich in pro-angiogenic and osteogenic miRNAs, significantly promote MSC osteogenesis and endothelial cell angiogenesis *in vitro*, and effectively drive bone regeneration in a rat cranial defect model (Fig 10A).

Pang et al. explored the use of BMSC-derived CDNs for the treatment of OA [185]. The authors demonstrated that BMSC-generated CDNs exhibited similar size compared to cell-secreted EVs, whilst the CDNs were produced at a 100-fold higher yield. They reported that the BMSC-CDNs promoted the proliferation, migration and differentiation of recipient chondrocytes and hBMSCs, in addition to stimulating the polarisation of macrophages towards an M2 phenotype. Within a mouse OA model, BMSC-derived CDNs delivered within GelMA ameliorated OA severity, decreased catabolic factor secretion and enhanced matrix synthesis (Fig 10B). D’Atri et al. similarly demonstrated the potential of MSC CDNs for OA, where the authors observed cartilage tissue targeting *in vitro* and *in vivo*, whilst *in vivo* studies reported the modulation of inflammatory processes, slowing down cartilage degradation in a mouse model of instability-induced OA [181]. Taken together, these studies highlight the potential of CDNs as EV-mimetic drug delivery system for orthopaedic regeneration. Due to the potential enrichment of cellular components (i.e. DNA) within the CDNs, however, thorough assessment on the safety of these nanoparticles is required prior to clinical application.

### Hybrid EVs

Hybrid EVs are formed by combining EVs and liposomes, offering the potential amalgamation of benefits from both systems, encompassing enhanced loading capacity, ease of synthesis, improved biocompatibility, increased safety, reduced immune response, and potential tissue-targeting capacity [193, 194]. This method enables the incorporation of diverse molecules, including antibodies, peptides, probes, fluorescent tags, and therapeutic agents - directly into the hybrid EV structure, thereby significantly enhancing their capabilities [195].

Hu et al. developed a novel hybrid nanoparticle strategy for targeted bone regeneration [168]. They achieved this by genetically overexpressing CXCR4 in NIH-3T3 cells, yielding EVs that displayed CXCR4 on their surface. These CXCR4-positive EVs were then fused with liposomes carrying antagomir-188 to create the hybrid nanoparticles. This engineering approach enabled specific accumulation of the nanoparticles within the bone marrow, due to the high local expression of SDF-1α, the ligand for CXCR4, where they effectively promoted BMSC osteogenesis and alleviated age-related bone loss in mice. This approach highlights the potential of hybrid EV-liposome systems for targeted delivery of therapeutic cargo to bone. In another study, Liu et al. developed bone-targeting hybrid EVs for alveolar bone regeneration [196]. The authors generated hybrid EVs by co-extruding MSC-derived EVs with osteogenic peptide (DSS)-modified liposomes. This strategic hybridization confers specific bone-targeting capabilities via the DSS peptide’s affinity for hydroxyapatite, while harnessing the inherent pro-osteogenic and pro-angiogenic benefits of MSC-EVs. This engineered system effectively promotes osteogenic differentiation and angiogenesis *in vitro*, significantly enhancing alveolar bone regeneration in an *in vivo* defect model.

Chen et al. designed a novel hybrid EV to specifically target chondrocytes and activate FGF18 gene expression [197]. The authors engineered hybrid EVs by fusing CAP-functionalised EVs with a liposome, loaded with SgFGF18. The gene-editing tool, CAP/FGF18-hyEVs, effectively activated FGF18 expression in human OA chondrocytes. When delivered within HAMA microgels, these hybrid EVs promoted chondrocyte proliferation and ECM production by modulating PI3K/AKT signalling.

### EV-inspired liposomes

Liposomes currently represent the most extensively utilized nanotechnology for the fabrication of synthetic EV-mimicking nanoparticles, capitalizing on their shared fundamental structure of a vesicular lipid bilayer. Employing liposomes as a foundational platform for generating synthetic EVs offers distinct advantages, including: 1) precise control over their biochemical composition, 2) the potential for scalable manufacturing, and 3) relative ease in achieving pharmaceutical-grade quality [198]. However, the rational design of truly EV-inspired liposomes hinges on the continued elucidation of the specific bioactive molecules within native EVs, responsible for their therapeutic mechanisms, enabling the recapitulation of these functionalities in EV-inspired liposomes.

BMP2 serves as a prime example of a therapeutic cargo that is explored for delivery via liposomes. While clinically employed to treat non-union fractures, the supraphysiological doses often required for efficacy can lead to significant adverse effects, such as hematoma, myelopathy, inflammation, and heterotopic ossification [199]. Consequently, the encapsulation of BMP2 within liposomes has been investigated as a strategy to enhance its localised delivery and potentially reduce systemic side effects. For instance, Crasto et al. incorporated recombinant human BMP2 (rhBMP-2) into liposomes that exhibited controlled release upon ultrasound stimulation, demonstrating ectopic bone formation *in vivo*, albeit without a direct comparison to the effects of the drug alone [200]. In another study, Liu et al. developed adhesive liposomes, loaded with BMP2 and delivered them via an injectable PEG hydrogel to osteoporotic femoral fracture sites in rats over an 8-week period [191]. Compared to non-adhesive controls, these adhesive liposomes exhibited enhanced tissue adhesion, resulting in improved osteogenic differentiation and accelerated bone remodelling at the fracture site (Fig 10C). Beyond the delivery of pre-existing biological factors, researchers are also exploring the potential of the liposomal membrane itself to elicit therapeutic effects, mirroring the inherent bioactivity of the EV membrane. Cui et al. developed ‘sterasomes,’ a liposomal formulation composed of the osteoinductive oxysterol 20S-hydroxycholesterol and the cationic amphiphile stearylamine [201]. These synthetic vesicles were shown to induce osteogenic differentiation of murine BMSCs *in vitro* without the need for additional therapeutic molecules. To achieve active targeting to bone tissue, Tao et al. formulated acid oligopeptide-modified liposomes using Glu6, a representative acid oligopeptide known for its high binding affinity to calcium ions in hydroxyapatite [202]. Among the tested concentrations, liposomes modified with Glu6 at 5 mol% exhibited the highest bone-targeting efficiency.

For cartilage repair, liposomes enriched with the pro-chondrogenic molecule KGN were fabricated. This study introduces a strategy for targeted cartilage repair using KGN encapsulated in liposomes and surface-modified with alkylated chondroitin sulphate (CS). The researchers demonstrated that hydrophobic modification of CS allowed interaction with liposomal membranes. While alkylated CS alone was cytotoxic, coating KGN-loaded liposomes with a specific alkylated CS derivative mitigated this. Notably, these KGN-loaded, CS-coated liposomes enhanced chondrogenic marker expression in mesenchymal stem cells and reduced hypertrophic tendencies compared to the polymer alone, suggesting a promising nanoformulation for cartilage regeneration [203]. In another study, with the A2A receptor as a key protein in the maintenance of cartilage homeostasis, liposomes were used as vectors for the delivery of either Adenosine or CGS21680, a selective A2AR agonist. Applied to both a murine model of obesity-induced OA and a rat model of post-traumatic OA, both formulations were found to promote cartilage formation *in vivo* and established A2AR as a novel target against OA [192] (Fig 10D). TGF-β1 is a potent growth factor demonstrated to have positive effects on chondrocytes, however, due to its low stability, half-life and poor permeability in cartilage, its application has been limited. Velot et al. employed agro-based rapeseed liposomes to encapsulate TGF-β1 and improve the growth factor stability, half-life and tissue permeability [204]. The authors showed that the liposomal delivery of TGF-β1 activated the ERK/p-38 MAPK/Smad signalling pathway, thus maintaining rat chondrocyte functionality *in vitro*. These studies collectively highlight the significant potential of liposomes as EV-mimetics for orthopaedic regeneration. This strategy hinges on identifying the key bioactive molecules responsible for the diverse functions of native EVs and then incorporating these into the liposomal mimetic.

Given the broad range of biological cargo within EVs that confers holistic therapeutic functions - including tissue targeting, immune regulation, and cellular differentiation - researchers face the challenge of determining the optimal degree of biomimicry necessary for clinical efficacy. Moreover, although EV-mimetic systems offer scalable and controllable alternatives to natural EVs, several limitations remain. Top-down approaches, such as cell extrusion, may lead to heterogeneous vesicle populations and membrane damage, reducing biological fidelity. Bottom-up synthetic systems, while allowing precise compositional control, often lack the complex functional biomolecules and targeting capabilities of natural EVs. Additionally, standardization and reproducibility across fabrication methods remain major challenges, and potential immunogenicity or cytotoxicity of synthetic components requires careful evaluation. Addressing these issues is essential for translating EV-mimetic systems into clinically viable platforms. These distinctions are summarized in Table 3, which highlights differences in characteristics, preparation methods, advantages, and limitations relative to natural EVs.

**Table 3:** Overview of key differences, advantages and disadvantages of EV-mimetic systems

EV-mimetic	Characteristics	Preparation method	Advantages	Limitations
CDNs	Retain cell membrane proteins and cytosolic cargo; similar EV size	Mechanical extrusion, sonication, chemical disruption	Scalable production; partially preserves native EV functionality	Potential heterogeneity; possible membrane damage
Hybrid EVs	Fusion of natural EVs with synthetic liposomes; hybrid membrane composition	Membrane fusion, extrusion, blending	Combines EV bioactivity with enhanced stability, targeting, or cargo loading	Complex preparation; regulatory challenges
EV-inspired liposomes	Fully synthetic; mimic EV size, lipid composition, and targeting properties	Lipid film hydration, extrusion, microfluidics	High reproducibility; customizable cargo and targeting	Lack natural EV biomolecules; may not fully replicate bioactivity

## EV-functionalised biomaterials for regenerative orthopaedics

EVs have gained attention as promising mediators in regenerative medicine and drug delivery [51]. However, their therapeutic potential is often hindered by rapid clearance, low stability, and inefficient targeting [205, 206]. While injection-based delivery of EVs in saline is accessible and effective for most musculoskeletal locations, there is limited control in the release kinetics, often resulting in rapid clearance rates and suboptimal regeneration. This necessitates frequent administrations to maintain clinical relevance, which can be burdensome for patients and increase the risk of infection, complicating its clinical translation [207]. To address these challenges, biomaterial-based approaches have been increasingly explored as a method to improve the bioavailability and thus EV regenerative capacity for bone and cartilage regeneration [31, 208, 209]. Due to the unique biophysical properties of bone and cartilage tissues, the use of appropriate biomaterial systems tailored for the tissue of interest is crucial for effective EV-induced regeneration.

### EV-biomaterial systems for bone repair

The inherent versatility of the EV surface offers numerous opportunities for functionalisation with biomaterial systems [31], a strategy increasingly employed to enhance their sustained release and thus their therapeutic efficacy in orthopaedic applications. This has spurred extensive investigations to integrate EVs with a diverse range of biomaterials for bone regeneration. Biomaterials for bone repair must achieve an optimal balance between mechanical strength and bioactivity to support load-bearing functions while promoting osteogenic differentiation and mineralized matrix formation. An emerging design principle emphasizes the rational selection of biomaterial systems based on the mechanical, biological, and release requirements of the target tissue. Recently, researchers have harnessed the unique physicochemical properties of EVs to develop strategies that control their release kinetics *in vivo* [210]. These approaches include physical immobilization of EVs within scaffolds, incorporation of ECM-binding motifs, and modulation of electrostatic interactions to achieve sustained and localized delivery [51].

Hydrogels have emerged as particularly adaptable platforms for the controlled delivery of EVs. These 3D hydrophilic polymer networks, capable of retaining significant amounts of water while maintaining structural integrity, exhibit excellent biocompatibility, rendering them well-suited for biological applications [211, 212]. Their inherent porous architecture allows for the physical encapsulation of EVs, facilitating localised and sustained release, which can significantly improve EV bioavailability and therapeutic impact [213]. Li et al. combined MSC-EVs with the commonly utilized GelMA hydrogel, which serves as a biocompatible, injectable scaffold that effectively physically encapsulates and retains EVs at the defect site, enabling sustained release [214]. The authors showed that the GelMA EV system enhanced osteogenesis and modulated immune responses by promoting macrophage polarization toward an M2 phenotype. While this strategy shows promise, biomaterials that degrade rapidly may compromise defect stabilization, which is crucial for the repair of load-bearing tissues.

Beyond the physical EV immobilisation, there has been growing research to further control EV release kinetics, maximising therapeutic potency. Owing to the inherently negative surface charge of EVs, researchers have investigated leveraging electrostatic interactions to enhance EV binding to biomaterial systems [215]. Synthetic nanoclays, such as laponite, have been shown to exhibit a broad affinity for bioactive molecules, attributed to their positively charged rims and negatively charged surfaces [216]. For instance, Man et al. demonstrated that combining epigenetically activated osteoblast-derived EVs with a GelMA/nanoclay composite hydrogel improved both EV release kinetics and osteoinductive potency [217]. Notably, the inclusion of nanoclay enhanced the hydrogel’s biomechanical properties, including compressive strength, rheological behaviour, and 3D printing fidelity, while also exhibiting a dose-dependent effect on EV release. Importantly, these epigenetically primed EVs within the hydrogel significantly enhanced the recruitment, epigenetic activation, and osteogenic differentiation of hBMSCs. A recent study built on this approach harnessing the positively charged polymer chitosan to control the delivery of EVs. The authors investigated developing a chitosan-based EV-capturing scaffold for the *in situ* enrichment of EVs via lipophilic and electrostatic interactions [218]. The authors designed a chitosan hydrogel, functionalised with phosphatidylserine-binding peptides to selectively capture endogenous neutrophil-derived EVs at bone defect sites. This EV-capturing scaffold promotes rapid vascularisation and enhances osteogenesis without requiring exogenous cell or EV loading. In a critical-sized cranial defect model, this approach accelerated angiogenesis and modulated the immune microenvironment to support robust bone regeneration.

**Fig. 11:**
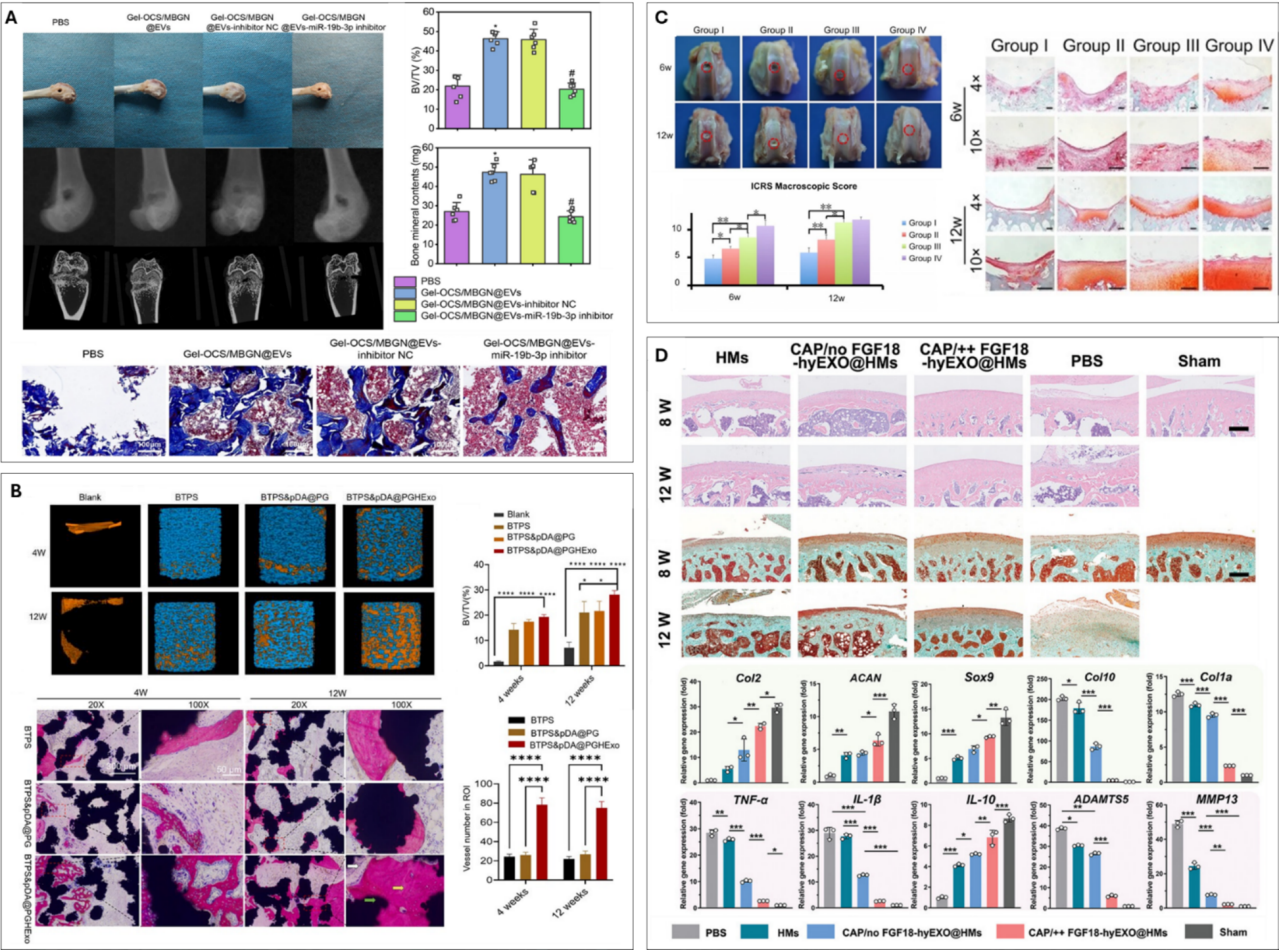
EV-functionalised biomaterials for orthopaedic regeneration. (**A**) macroscopic, X-ray and µCT images of new bone formation at the site of a femoral defect. BV/TV and bone mineral content analysis. masson staining for collagen fiber formation in each group. adapted from [220] under the creative commons license, 2023. (**B**) 3D µCT images, BV/TV% analysis and sectional views demonstrating bone ingrowth within the defect regions for each scaffold type. methylene blue/fuchsin-stained sections. vessel counts across scaffold groups. adapted from [222] under the creative commons license, 2025. (**C**) macroscopic evaluation of the osteochondral defect regions at 6 and 12 weeks, ICRS macroscopic scores and safranin O staining of tissues. adapted from [223] under the creative commons license, 2019. (**D**) H&E and safranin O/fast green staining. the gene expressions related to cartilage ECM, fibrous and hypertrophic cartilage, matrix degradation, pro-inflammation and anti-inflammation. adapted from [197] with permission from John Wiley and Sons, 2024

EVs have been shown to interact with native ECM components of the bone, offering a biomimetic strategy to localize and enhance the delivery of pro-regenerative vesicles for bone repair [219]. Thus, hydrogels consisting of ECM components have also been investigated to effectively deliver EVs. For instance, researchers developed an ECM-mimetic chitosan/collagen composite hydrogel to effectively deliver osteoblast EVs for bone regeneration. The authors exploited electrostatic interactions to capture the negatively charged EVs with the positively charged chitosan, whilst the EV-associated integrins bound to collagen. The authors show that ECM-mimetic composites enhanced EV controlled release kinetics and promoted EV-induced bone-like tissue formation [213]. In another study, researchers developed a biomimetic composite hydrogel, consisting of oxidized chondroitin sulphate (OCS), mesoporous bioactive glass nanoparticles (MBGNs) and gelatine, designed to enhance BMSC EV-induced bone regeneration [220]. The resulting Gel-OCS/MBGN@EVs hydrogel acts as a sustained release platform for EVs, which delivered miR-19b-3p to target cells. This miR-19b-3p then suppresses the expression of WWP1 (WW domain–containing E3 Ub protein ligase 1), a negative regulator of osteogenic differentiation, ultimately accelerating new bone formation and femoral defect repair in rats (Fig 11A). These studies demonstrate how chemical and structural compatibility between biomaterial and EV surface molecules informs release control and downstream biological outcomes.

Beyond hydrogels, other biomaterial scaffolds have shown promise. A recent study by Sun et al. incorporated hUC-MSCs EVs into a 3D-printed silk fibroin/collagen I/nano-hydroxyapatite scaffold, creating a cell-free bone tissue engineering system that effectively stimulated rat alveolar bone defect healing and angiogenesis [221]. Luo et al. investigated a dual-biomimetic strategy for bone regeneration, harnessing 3D-printed titanium trabecular scaffolds (BTPS) loaded with hypoxia-induced EVs (H-EVs) [222]. H-EVs, derived from HUVECs and encapsulated within a PEGDA/GelMA hydrogel microspheres and anchored onto BTPS using polydopamine (pDA) modification (BTPS&pDA@PGH-EVs), significantly enhanced osteogenesis and angiogenesis *in vitro* via MAPK, mTOR, HIF-1, and VEGF pathways. *In vivo*, the BTPS&pDA@PGH-EVs composite markedly improved bone volume, density, and neovascularization in a rabbit model, offering a promising solution for personalized bone defect repair (Fig 11B).

Comparative analyses across these studies reveal that EVs derived from different cell types, such as MSCs, osteoblasts, or endothelial cells, exert distinct regenerative effects that correlate with their intrinsic molecular cargo and biological roles. MSC-derived EVs generally promote osteogenesis and immunomodulation, whereas endothelial EVs primarily enhance angiogenic responses necessary for vascularised bone repair. This functional complementarity suggests that EV source selection should be tailored to the biomechanical and healing context, with composite biomaterials serving as tunable platforms to balance mechanical support and release duration.

Growing interest has focused on the incorporation of EVs into biomaterial platforms to enhance their local retention, bioavailability, and therapeutic efficacy. While early studies demonstrate encouraging outcomes, a deeper understanding is needed of how the biomaterial milieu modulates EV-mediated bone regeneration post-delivery. Furthermore, defining optimal *in vivo* dosing strategies remains essential - not only to maximize therapeutic benefit but also to inform scalable manufacturing protocols, tailored to specific clinical applications. Several limitations of current preclinical models hinder clinical translation. Most studies employ small-animal defect models that do not replicate the mechanical, immunological, and vascular complexities of human bone. Moreover, inconsistencies in EV isolation, dosing, and release kinetics hinder direct comparison and reproducibility. Moving forward, the field would benefit from standardized in vivo models, large-animal validation, and systematic evaluation of how biomaterial degradation dynamics influence EV bioactivity and safety. These efforts will be essential to define scalable, clinically compliant design principles for EV-biomaterial systems in regenerative orthopaedics.

### EV-biomaterial systems for cartilage repair

Achieving successful cartilage regeneration necessitates design of biomaterials with distinct mechanical properties compared to those employed for bone repair. Specifically, overly rigid scaffolds can impede chondrogenesis and, critically, induce undesirable endochondral osteogenesis, where cartilage is prematurely or inappropriately converted into bone [15, 224]. Consequently, biomaterials for cartilage repair must balance structural integrity with sufficient compliance to preserve the chondrocyte phenotype and facilitate ECM deposition. Hydrogels with their tuneable viscoelasticity and high water content, closely mimic the native cartilage ECM and are therefore the most widely explored platforms in EV-based cartilage tissue engineering. Emerging design principles emphasize the co-optimization of EV source, matrix stiffness, and degradation rate to achieve synchronized release kinetics with the temporal sequence of cartilage regeneration. In this context, incorporating ECM proteins has emerged as the most common strategy, enabling researchers to synergistically regulate EV release kinetics while supporting de novo tissue regeneration.

Particularly, ECM-derived proteins found within native cartilage such as collagen, fibronectin, sulphated glycosaminoglycans, hyaluronic acid have been incorporated into biomaterial systems to facilitate EV loading and enhance bioactivity for cartilage repair [225]. For instance, Chen et al. developed a GelMA/ECM scaffold for the delivery of MSC-derived EVs [223]. The authors combined decellularised porcine cartilage with GelMA and murine MSC EVs, demonstrating the successful 3D printing of this bioink into radially oriented 3D architectures, exhibiting a sustained release of EVs *in vitro*. Moreover, the EV-functionalised material effectively enhanced cellular migration, supporting the polarisation of synovial macrophages to the M2 phenotype, facilitating the regeneration of osteochondral defects in rabbit (Fig 11C). Liu et al. explored the impact of encapsulating human induced pluripotent stem cell-derived EVs (hiPSC-EVs) within a light-sensitive hydrogel glue (composed of o-nitrobenzyl alcohol moieties-modified hyaluronic acid and gelatine) for articular cartilage regeneration [226]. Their findings demonstrated that this EV-functionalised hydrogel significantly promoted chondrocyte proliferation *in vitro* and substantially enhanced articular cartilage repair in a rabbit defect model compared to delivering hiPSC-EVs in solution. In another study, Xing et al. developed an injectable, thermosensitive porcine nucleus pulposus dECM hydrogel, functionalised with rat ADSC-EVs (dECM@EVs) to treat intervertebral disc degeneration (IVDD) [227]. The dECM@EVs hydrogel provides sustained EV release, which in turn regulates NPC metabolic balance (matrix synthesis/degradation) and inhibits pyroptosis, thus ameliorating IVDD in rats. Chen et al. developed an innovative injectable microgel system for OA therapy, fundamentally built upon methacrylic anhydride-modified hyaluronic acid (HAMA) hydrogel microspheres [197]. This biomaterial serves a dual purpose: first, as a platform for the localised delivery of hybrid EVs (CAP/FGF18-hyEVS), which are engineered with a CAP and carry a CRISPR/Cas9-based tool for targeted FGF18 gene activation in chondrocytes. Second, the HAMA microgel itself provides self-renewable joint lubrication, directly addressing friction in the joint. This biomaterial-driven approach synergistically promotes cartilage regeneration, reduces inflammation, and prevents ECM degradation in OA (Fig 11D).

Physical encapsulation of EVs has been investigated to sustained EV release for cartilage repair. For instance, In a recent study, Liang et al. harnessed microalgae (*Spirulina platensis*) as an alternative source of EVs for OA treatment [230]. The microalgae EVs, rich in bioactive metabolites, modulated mitochondrial function and promoted cellular energy homeostasis in chondrocytes. When combined with an anti-inflammatory rhein hydrogel, the EV-hydrogel synergistically reduced inflammation, prevented cartilage degradation, and restored joint metabolism in a DMM-induced OA model in mice. In another study, Tao et al. developed an injectable, thermosensitive hydrogel (PDLLA‑PEG‑PDLLA, PLEL) to physically entrap EVs derived from synovial MSCs overexpressing circRNA3503 [146]. This system enabled sustained local release of EVs, which promoted cartilage matrix synthesis, inhibited ECM degradation, and protected chondrocytes from apoptosis. *In vivo*, intra-articular injection of the hydrogel-EV combination significantly preserved cartilage structure in an OA model, highlighting its potential for targeted cartilage repair.

Electrostatic interactions have emerged as an effective strategy to enhance the retention and controlled release of EVs from biomaterial systems for tissue repair. Leveraging this approach, Hu et al. investigated delivering hUC-MSC EVs with a GelMA/nanoclay hydrogel to improve cartilage regeneration [229]. The authors showed that the incorporation of nanoclay substantially sustained the release of hUC-MSC EVs compared to GelMA alone. The delivery of the EV-functionalised GelMA/nanoclay hydrogel improved cartilage regeneration in rats via inhibiting reducing tensin homolog deleted on chromosome 10 (PTEN) and phosphatase expression. Furthermore, it was revealed that EV-associated miR-23a-3p increased the expression of protein kinase B, which promoted migration, proliferation and differentiation of chondrocytes and MSCs *in vitro*.

Comparative analysis across these studies reveals distinct structure-function relationships between EV source, bioactive cargo, and biomaterial platform. MSC- and ADSC-derived EVs primarily enhance chondrocyte proliferation and matrix synthesis, whereas iPSC-EVs provide higher regenerative plasticity but raise challenges regarding production consistency. Non-mammalian EVs, such as those from microalgae offer a renewable and ethically unencumbered source [228, 229], however, require rigorous validation to confirm biocompatibility and functional equivalence. These findings underscore that optimal outcomes in EV-assisted cartilage regeneration arise from aligning EV source characteristics with the mechanical, biochemical, and temporal requirements of the chosen biomaterial system.

Although there has been progress in developing EV-functionalised biomaterials for cartilage repair, future research must thoroughly investigate the interplay between biomaterial degradation and EV release kinetics, critically defining the optimal timing and dosage for therapeutic efficacy. Moreover, most current preclinical studies are largely confined to small-animal models that fail to replicate the complex biomechanical loading, zonal organization, and inflammatory microenvironment of human cartilage. Moreover, differences in EV isolation, characterization, and dosing complicate cross-study comparison. Future progress will require standardized evaluation frameworks, advanced large-animal models, and scalable, good manufacturing practice (GMP)-compliant production of both EVs and their carrier matrices. Addressing these translational gaps will be essential to realise the clinical promise of EV-functionalised biomaterial systems for cartilage repair.

## Challenges and future perspectives

EVs have intrigued the scientific community for over five decades for their diagnostic and therapeutic promise; however, their full potential is only now being realised, as growing evidence positions EVs as a powerful and versatile class of bioactive materials, evident by 258 EV-based therapeutic interventions currently registered on ClinicalTrials.gov (search terms: “extracellular vesicles” OR “exosome”). Table 4 provides an overview of clinical trials registered for EV-based treatments for skeletal medicine, underscoring a concerted global effort and growing confidence in the feasibility of clinical translation. Moreover, the global EV market is experiencing a rapid expansion due to technological advancements in EVs isolation and analysis, and a broad range of clinical applications (including cancer, cardiovascular, inflammatory, and neurodegenerative diseases) which has led to increasing public and private sector investments. The global EV market size was estimated at 177.4 million USD in 2024 and is projected to reach 794.2 million USD by 2030, growing at a compound annual growth rate of 28.73% from 2025 to 2030 [231]. While the clinical translation of EVs shows immense promise, significant challenges remain in the field that must be addressed to ensure their successful and timely progression to clinical application.

**Table 4:** Clinical trials of EV-based treatments for orthopaedic disorders

Identifier (sponsor)	Specific disease	Study phase	Study design	Last update
NCT04998058 (Pontificia universidade Católica do rio grande do Sul)	Bone loss	Phase 1/2 (not yet recruiting)	Bone augmentation at the floor of the maxillary sinus with bone substitutes combined with MSC EVs	06–2025
NCT04849429 (Dr. Himanshu Bansal Foundation)	Degenerative disc disease	Phase 1 (completed)	Intra-discal injection of autologous platelet-rich plasma (PRP) enriched with exosomes (PRPEX) in chronic low back pain	07–2022
NCT04281901 (university medical centre Ljubljana)	Bone inflammation	Phase 1 (completed)	Platelet- and EV-rich plasma for the treatment of chronically inflamed post-surgical temporal bone cavities	08–2021
NCT05520125 (institute of biophysics and cell engineering of national academy of sciences of belarus)	Segmental fracture, bone loss	Phase 1/2 (not yet recruiting)	Treatment of patients with segmental bone tissue defects using mesenchymal stem cells enriched by EVs	08–2022
NCT06463132 (Rion Inc.)	OA	Phase 1 (recruiting)	intra-articular injections of purified exosome product (PEP) at a low dose (one vial PEP) and high dose (two vials PEP), with and without EUFLEXXA (sodium hyaluronate), for the treatment of Knee OA	06–2024
NCT06431152 (universidad de los Andes)	OA	Phase 1 (recruiting)	intra-articular injections of exosomes (sEVs) from allogeneic UC- MSCs delivered in the knee of patients with mild to moderate symptomatic OA	05–2024
NCT06937528 (university of Jordan)	OA	Phase 1 (recruiting)	Intra-articular EV injection in the knees of patients with advanced stage III and IV OA	04–2025
NCT05060107 (Francisco Espinoza)	OA	Phase 1 (unknown status)	Intra-articular injection of MSC Exosomes (CelliStem®OA-sEV) in patients with moderate Knee OA (ExoOA-1)	09–2021

EVs confers distinct therapeutic advantages over traditional approaches. In comparison, platelet-rich plasma (PRP), though widely used and clinically accessible, often exhibits variability in composition and inconsistent therapeutic outcomes due to donor-dependent differences and limited control over bioactive factor release. EVs, by contrast, provide a more defined and tunable therapeutic platform, allowing for engineering of their content and surface properties to enhance tissue-specific targeting and regenerative outcomes. Nonetheless, several translational barriers persist, including challenges in standardizing isolation methods, ensuring batch-to-batch reproducibility, and navigating regulatory frameworks that distinguish EVs from conventional biologics. Addressing these issues will be crucial for realizing the full clinical potential of EV-based therapies and positioning them as a complementary or next-generation alternative to established modalities such as PRP.

Acquiring a mechanistic understanding of how EV-based therapies induce tissue healing, is crucial for both regulatory approval and onward clinical implementation [232]. While single-omics analyses have provided initial insights into EV biology, they inherently fall short of capturing the intricate interplay between nucleic acids, proteins, lipids, and metabolites that underpins complex biological processes. A comprehensive understanding of EV functions, origins, and therapeutic applications in regenerative orthopaedics therefore critically necessitates an integrative multi-omics approach to unravel these synergistic molecular networks [233]. Multi-omics integration, encompassing proteomics, transcriptomics, lipidomics, and metabolomics, can reveal key molecular signatures of EV potency, elucidate intercellular signaling mechanisms, and support the identification of biomarkers predictive of therapeutic outcomes. Such systems-level analyses will also aid in defining critical quality attributes essential for standardization and regulatory compliance.

A crucial frontier in EV research lies in harnessing advanced computational and artificial intelligence (AI)-driven methods to analyse and interpret complex multi-omics datasets. Machine learning and deep learning algorithms can integrate heterogeneous data types to map molecular interdependencies, identify novel EV subpopulations, and predict functional outcomes based on cargo composition [234]. The integration of AI-based analytics with EV characterization provides a more precise molecular fingerprint of EVs, enabling their use as powerful diagnostic and therapeutic tools. Furthermore, computational algorithms facilitate the discovery of EV source–function relationships, discrimination of vesicle subtypes, and prediction of their regenerative potential, offering unprecedented resolution in understanding EV biology [235]. In the context of biomanufacturing, AI-driven quality control systems offer exciting possibilities for improving consistency and reproducibility. Real-time monitoring of production parameters and EV characteristics through predictive models could allow early detection of batch deviations or contamination, ensuring process robustness and compliance with GMP standards. Such tools will be pivotal for closed-loop process control, reducing human error, and accelerating regulatory approval by providing traceable, data-rich validation of EV product integrity.

Harnessing EV-based technologies as therapeutics offers several compelling advantages; however, successful clinical translation necessitates strict adherence to GMP. The inherent biological complexity of EVs, coupled with natural batch-to-batch variations during production, introduces greater manufacturing risks compared to synthetic nanomedicines [235]. A critical bottleneck hindering the widespread clinical translation of EV-based products, is the ability to achieve rapid, cost-effective, and reproducible large-scale production. Addressing this challenge requires the adoption of clinical-grade chemically defined media and serum replacements to eliminate xenogeneic contaminants. The implementation of bioreactor systems for the scalable production of clinical grade MSC-derived EVs represents a significant step towards translational feasibility [236].

Moving forward, innovations such as automated downstream purification, tangential flow filtration, and microfluidic isolation will further enhance scalability and purity [237, 238]. Equally important is the development of robust potency assays and stability testing frameworks to ensure long-term product safety and functional retention during storage and distribution. Rigorous quality assessment and batch-to-batch variability evaluation of batch-to-batch variability are essential to meticulously monitor the consistency maintain reproducibility of the isolated EV population throughout the scale-up process [239]. Ultimately, the convergence of multi-omics, AI-driven analytics, and GMP-compliant biomanufacturing will define the next generation of EV-based therapeutics. By integrating systems biology with data science and process engineering, the field can transition from empirical development to rational, standardized, and predictive EV design, paving the way toward safe, effective, and clinically viable regenerative therapies.

The progression of EV-based therapies in regenerative orthopaedics is further constrained by heterogeneous reporting and a deficit of standardisation in the existing literature. While the emergence of public databases like EV-TRACK, EVpedia, and ExoCarta has begun to address transparency and facilitate biomarker identification, consistent adherence to consensus guidelines, such as MISEV2023 [32], remains critical. Embracing such standards will be pivotal for both fostering scientific reproducibility and effectively educating the next generation of researchers in this rapidly evolving field.

Furthermore, the optimal storage of EV therapeutics remains a critical consideration. While stability at 4 °C or −80°C has been reported [240], alternative methods like lyophilisation are also explored. Notably, Jones et al. demonstrated that ADSC EVs retained their purity, size, and morphology upon rehydration after lyophilisation, even showing enhanced efficacy after two months compared to those stored at −80°C [241]. However, current storage studies predominantly focus on short-term physicochemical properties Future research should focus on long-term functional stability under precisely defined storage conditions, including freeze-thaw cycles, duration, temperature, and storage buffers, to ensure preserved therapeutic efficacy [242].

A significant safety concern associated with cell-derived nanoparticles is the potential presence of contaminants. Viruses, such as HIV or hepatitis C, could co-isolate with EVs. While radiation has been proposed as a potential sterilisation method, the lack of comprehensive data on potential collateral EV damage necessitates further investigation [243]. Overcoming these safety hurdles demands the development of standardised isolation and characterisation protocols, coupled with the implementation of robust quality control systems [244]. In parallel to refining native EV production and handling, the development of EV-mimetic systems offers a promising alternative to circumvent some of these translational challenges. Key aspects requiring thorough evaluation for these synthetic systems include physicochemical characterisation, biocompatibility and nanotoxicology, pharmacokinetics and pharmacodynamics, process control, and scale-up reproducibility [245].

Native and bioengineered EVs offer complementary approaches to regenerative medicine. Complex EV engineering, including cargo modification, surface functionalization, or membrane hybridization, allows for precise tuning of therapeutic properties, such as enhanced tissue targeting or controlled release of bioactive molecules. However, this approach can involve challenging fabrication processes, potential alteration of native EV composition, and regulatory complexity [246]. In contrast, biomaterial-based strategies, such as incorporating EVs into hydrogels, scaffolds, or nanoparticles, primarily improve stability, localized delivery, and sustained release, but may provide less molecular-level customization compared with direct EV engineering. Emphasizing EV-based nanomedicine is warranted as EVs inherently combine biological signaling capacity with nanocarrier versatility, bridging natural regenerative cues with controlled delivery platforms. Notably, even after extensive engineering, EVs generally retain at least part of their intrinsic biological activity, such as immunomodulatory or pro-regenerative effects, while also serving as carriers for additional therapeutic cargo [247]. Thus, careful engineering can preserve essential EV functions while augmenting their utility as versatile delivery vehicles.

As highlighted in our review, there has been extensive research harnessing novel bioengineering strategies to enhance the therapeutic efficacy of EVs for the treatment of bone and cartilage disorders. Although their promise has been demonstrated, it is critical to ensure the robustness, reproducibility, and scalability of these approaches to facilitate clinical translation. Moreover, standardized regulatory frameworks and comprehensive long-term safety evaluations are essential to establish the reliability and clinical acceptability of these next-generation nanoscale therapeutics in regenerative orthopaedics.

Finally, within the specific context of orthopaedic applications, the limited translational relevance of current preclinical animal models poses an additional challenge. The predominant use of small animal models necessitates a critical evaluation of employing clinically relevant defect sites and/or larger animal models on a case-by-case basis to generate more robust preclinical evidence of therapeutic potency. Such advanced models would significantly bolster the translational potential of EV-based treatments for orthopaedic disorders [51].

In summary, advancing EV-based therapies in regenerative orthopedics requires an integrated approach encompassing multi-omics analyses, AI-driven characterization, GMP-compliant scalable production, rigorous quality control, and improved preclinical modelling. Addressing these challenges will not only enhance our understanding of EV biology but also accelerate the translation of EV-based nanomedicine as a next-generation regenerative therapy.

## Conclusion

EVs have been rapidly emerging as a potent nanotherapeutic for bone and cartilage regeneration. This review has detailed the expanding understanding of EVs’ intrinsic roles in bone and cartilage healing and showcased the critical impact of cutting-edge bioengineering approaches in optimizing their therapeutic efficacy for orthopaedic applications. Sustained progress in these areas will be pivotal in unlocking the full therapeutic potential of EV-based nanotherapeutics as viable clinical solutions for orthopaedic regeneration.

## Data Availability

No datasets were generated or analysed during the current study.
